# Epigenetic editing approaches maturity: AI-driven precision design, delivery innovation, and the road to clinical translation

**DOI:** 10.1186/s13148-026-02194-x

**Published:** 2026-07-09

**Authors:** Shiqi Chang, Di Lu, Yuyuan Jin, Yilei He, Yue Zhao, Xinyu Xie, Jingjing Guo, Wenbing Xie, Yong Tao

**Affiliations:** 1 Center for Life Sciences, Yunnan Key Laboratory of Cell Metabolism and Diseases, School of Life Sciences, Yunnan University, Kunming, 650504 China; 2 Faculty of Applied Sciences, Centre in Artificial Intelligence Driven Drug Discovery, Macao Polytechnic University, Macao, 999078 China; 3 Department of Stomatology, Division of Life Sciences and Medicine, Hefei National Research Center for Interdisciplinary Sciences at the Microscale, School of Basic Medical Sciences, The First Affiliated Hospital of USTC, University of Science and Technology of China, Hefei, 230026 China

**Keywords:** Epigenetic editing, Gene silencing, DNA methylation, CRISPRoff, Lipid nanoparticle, Artificial intelligence, de novo protein design, Clinical translation

## Abstract

Epigenetic editing achieves durable gene silencing through targeted modification of chromatin and DNA methylation states without altering the genomic sequence—modifications that remain fundamentally reversible compared with genome editing. Long constrained by transient efficacy, insufficient precision, and delivery bottlenecks, the field reached a critical inflection point in 2024–2025, measurable through three quantifiable criteria: (1) mechanistic durability—silencing maintained across ≥ 450 cell divisions in vitro and ≥ 12 months in vivo without continued editor expression; (2) delivery competence—tissue-selective transduction at > 50% efficiency in liver, muscle, and whole brain via engineered lipid nanoparticle and AAV platforms; and (3) clinical validation—advancement of multiple first-in-human trials. The inaugural trial in epigenetic editing was OTX-2002 (targeting MYC-driven malignancies, MYCHELANGELO study), initiated in October 2022, followed by TUNE-401 for chronic hepatitis B (Phase Ib, November 2024) and EPI-321 for facioscapulohumeral muscular dystrophy (Phase I/II, first patient dosed August 2025). Artificial intelligence contributes at distinct levels: deep learning platforms have directly accelerated clinical-stage LNP formulation screening and AAV capsid prediction; AlphaFold3 has optimized protein–DNA interaction validation without yet entering clinical programs; and the 2025 de novo design of DNA-binding proteins smaller than 65 amino acids represents a proof-of-concept breakthrough with zero clinical precedent. This review comprehensively analyzes epigenetic editing’s technological maturation, provides a tiered assessment of AI’s realized and anticipated contributions, critically evaluates the emerging clinical landscape, and identifies decisive unresolved challenges—including long-term stability in non-dividing cells, the lack of monitoring systems and intervention protocols needed to implement reversibility in clinical practice, and manufacturing access barriers. We argue that epigenetic editing is progressively establishing itself as a distinctive therapeutic modality characterized by durable efficacy and sequence-independent safety—reversibility as a theoretical safety mechanism has been validated preclinically, but the monitoring and intervention infrastructure required for its clinical implementation remains to be established. Long-term human validation remains the outstanding core question.

## Introduction

**Fig. 1:**
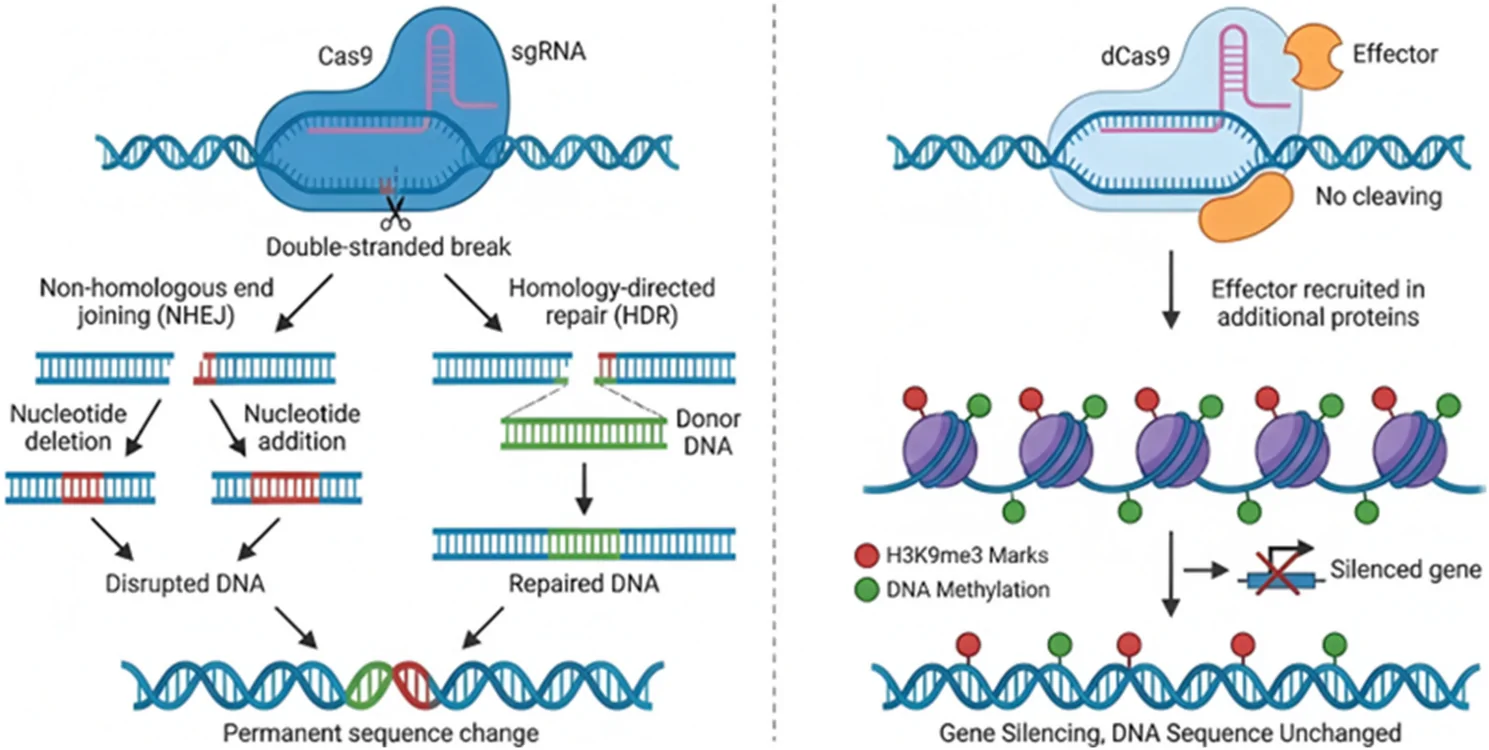
Mechanistic basis of genotoxicity risks in genome editing and avoidance strategies through epigenetic editing. Genome editing (left) relies on nuclease-mediated DNA double-strand breaks, with cellular repair pathways—non-homologous end joining or homology-directed repair—determining the final sequence outcome; this process is inherently stochastic and can generate large deletions, complex genomic rearrangements, and even chromothripsis, becoming irreversible once completed. Epigenetic editing (right) operates upstream of DNA sequence by recruiting effector domains through programmable DNA-binding domains (DNA methylation direction: DNMT or TET; chromatin remodeling direction: histone-modifying enzymes), establishing self-reinforcing, mitotically heritable chromatin states without introducing any strand breaks. The resulting gene silencing or activation combines durability with reversibility, providing a mechanistic alternative to the uncontrolled permanent sequence consequences of nuclease-based editing. DBD: DNA-binding domain; DSB: double-strand break; DNMT: DNA methyltransferase; TET: ten-eleven translocation enzyme

 Epigenetic editing represents a therapeutic paradigm capable of achieving durable gene regulation without permanently altering the genomic sequence. In 2023, CRISPR-Cas9 technology gave rise to the first approved CRISPR-based gene therapy, Casgevy [1], a landmark that was celebrated widely, yet the safety concerns arising from the irreversibility of genomic alterations have grown increasingly prominent. Studies have confirmed that CRISPR-Cas9 can induce large deletions, complex genomic rearrangements, and even chromothripsis closely associated with cancer [2, 3]. These findings profoundly underscore the necessity of developing alternative strategies—approaches capable of achieving therapeutic gene regulation without introducing permanent DNA alterations. The fundamental distinction between genome editing and epigenetic editing is illustrated in Fig. 1.

Epigenetic editing is a direct response to this challenge. It operates through the natural mechanisms of gene expression regulation, targeting DNA methylation and histone modifications [4–6], and thereby confers three core advantages: durability—epigenetic marks are faithfully transmitted during DNA replication [7, 8]; reversibility—gene silencing can be reversed through TET enzyme-mediated demethylation [9]; and differentiated safety characteristics—the silencing efficiency of epigenetic editors is broadly comparable to Cas9 gene inactivation at equivalent RNA doses [10], but any off-target perturbations are transcriptional rather than DNA sequence alterations, remain reversible, and completely circumvent the risks of large deletions, complex genomic rearrangements, and chromothripsis inherent to nuclease-based editing [2, 3, 11]. The field has undergone significant evolution: from first-generation CRISPRi systems requiring continuous expression [12, 13], to the CRISPRoff system capable of maintaining stable silencing across more than 450 cell divisions [11], and most recently to the compact CHARM system compatible with single-AAV delivery for whole-brain applications [14].

Before accurately assessing the role of artificial intelligence, it is necessary to provide an objective appraisal of its current translational status. The most clinically advanced epigenetic editing programs—Tune Therapeutics’ TUNE-401 [15] and Epicrispr Biotechnologies’ EPI-321 [16]—were designed using conventional molecular biology methods developed prior to 2021, temporally preceding the emergence of recent AI breakthroughs. The milestone of de novo-designed DNA-binding proteins achieved in 2025 [17], despite its substantial scientific significance, had not been integrated into any clinical candidate to date. This temporal gap does not represent failure; rather, it reflects the multi-year preclinical development cycle inherent to any therapeutic program. AI currently provides validated, clinically relevant contributions in three specific areas, distinguishable by tier: Tier 1, contributions that have directly advanced clinical-stage development—deep learning platforms have compressed LNP formulation screening from years of empirical exploration to months (AGILE [AI-Guided Ionizable Lipid Engineering] [18], COMET [Composite Material Transformer] [19], detailed in Section The AI revolution in lipid nanoparticle design), representing the currently most clinically proximal AI contribution; Tier 2, contributions that have optimized existing tools but have not yet been integrated into clinical programs—rational engineering of existing protein scaffolds yielding 11- to 100-fold activity improvements (AlphaFold3-guided Fanzor [20], NovaIscB [21], detailed in Section AI engineering optimization of epigenetic modifying enzymes and miniature editors), achievements that merit recognition, but whose tools are fundamentally gene-editing instruments, with relevance to epigenetic editing currently limited to exploratory fusion work; and Tier 3, contributions with breakthrough potential but zero clinical precedent—de novo designed DNA-binding proteins in 2025 (≤ 65 amino acids, 30–500 nM affinity) representing a proof-of-concept breakthrough, and rapid computational filtering of protein–DNA interactions by replacing low-throughput crystallographic screening [17], representing a proof-of-concept milestone still years from clinical application (both detailed in Section From structure prediction to de novo design: evolution of the AI protein design toolchain). The field awaits the first clinical program built around an AI-designed targeting module as a core component—the 2025 breakthrough in de novo DNA-binding protein design suggests this goal is achievable within this decade, but has not yet arrived.

This review operationalizes the core thesis of ‘approaching maturity’ through three quantifiable maturity criteria that run throughout the text: Criterion 1, mechanistic durability—maintenance of epigenetic silencing across ≥ 450 cell divisions in vitro or ≥ 12 months in vivo following transient editor expression, confirmed by quantitative methylation analysis [7, 8, 11]; Criterion 2, delivery competence—pharmacodynamically meaningful editing efficiency (target gene silencing ≥ 50%) in target tissues including those beyond the liver [14, 16]; Criterion 3, clinical validation—initiation of Phase I/II trials in humans with pre-specified pharmacodynamic endpoints [15, 16, 22]. Section Foundations of epigenetic editing technology assesses the molecular basis for Criterion 1, Section AI-enabled intelligent delivery systems maps delivery platform maturity to the degree of Criterion 2 satisfaction, Section Clinical translation progress: a critical assessment using the three maturity criteria as a framework systematically evaluates each clinical program’s evidence contribution to Criterion 3, and Section The non-dividing cell problem: a critical translational gap candidly acknowledges existing validation gaps for all three criteria in post-mitotic tissues.

## Foundations of epigenetic editing technology

Epigenetic editing has officially entered the phase of clinical validation. In August 2025, EPI-321—the world’s first epigenetic editing therapy for neuromuscular disease—was administered to a patient with facioscapulohumeral muscular dystrophy [16]. This is an important expansion of the clinical landscape of epigenetic editing following the first-in-human administration of OTX-2002 (MYCHELANGELO study) [22] in October 2022, marking the extension of this technology into the treatment of hereditary neuromuscular diseases. Behind this milestone lies nearly three decades of technological accumulation—from early explorations with zinc finger proteins, through the transitional breakthrough of transcription activator-like effectors, to the revolutionary transformation brought about by CRISPR/dCas9—completing the historical metamorphosis of epigenetic editing from ‘can it be done’ to ‘how can it be done better.’ Unlike conventional genome editing, epigenetic editing does not alter the DNA sequence; instead, it regulates gene expression bidirectionally through targeted rewriting of epigenetic marks at specific genomic loci—encompassing both gene silencing via DNA methylation and repressive histone modifications, and gene activation via histone acetylation and DNA demethylation [13, 23].

### Conceptual framework and core principles

Epigenetic editing is defined as the targeted rewriting of epigenetic marks at predetermined genomic loci through the fusion of programmable DNA-binding domains with epigenetic effector domains [24]. This definition encompasses two essential technical modules: the DNA-binding platform that addresses ‘where’ modifications are made, and the effector domain that determines ‘what’ modification is deposited. The fundamental distinction between epigenetic editing and conventional genome editing manifests in three core features—durability, reversibility, and differentiated safety characteristics—whose mechanistic basis has been articulated in the introduction, with clinical validation evidence to be systematically evaluated in Section The PCSK9 case: dual preclinical validation of durability and Reversibility.

### First-generation systems: the zinc finger protein era (1997–2009)

The conceptual origins of epigenetic editing trace back to 1997, when Guo-Liang Xu and Timothy H. Bestor first proposed targeting DNA cytosine methylation to predetermined sequences [25]. The realization of this vision depended on zinc finger proteins (ZFPs)—at the time the only available programmable DNA-binding platform, discovered by Miller, McLachlan, and Klug in 1985 [26]. Each zinc finger module (approximately 30 amino acids) coordinates a zinc ion through a Cys₂His₂ motif, forming a ββα fold whose α-helix contacts three consecutive base pairs in the major groove of DNA [27]. The tandem arrangement of multiple modules enables sequence-specific recognition, establishing ZFPs as the first-generation programmable DNA-binding platform.

In 2002, Snowden and colleagues provided a critical proof-of-concept: fusion of ZFPs with histone methyltransferases (catalytic domains of SUV39H1 and G9a) demonstrated that H3K9 methylation could be deposited at the endogenous VEGF-A locus to initiate gene silencing—the first cellular proof-of-concept for targeted histone modification-mediated gene repression [28]. Key contributors of this era included Marianne Rots, who pioneered and systematically defined the concept of ‘epigenetic editing’ [24]; Carlos Barbas III and David Segal, pioneers of modular ZFP design [29]; and Pilar Blancafort, who focused on epigenetic reprogramming of cancer cells [30]. Although the ZFP platform was gradually superseded by TALE and CRISPR systems after 2009, its conceptual legacy continued to generate important advances: Jeltsch’s group combined DNMT3A–DNMT3L single-chain fusion proteins with ZFPs, achieving targeted DNA methylation at the VEGF-A locus with approximately 10-fold higher methylation activity than DNMT3A alone [31]; Blancafort’s group extended ZFP-based epigenetic reprogramming strategies to cancer cell models [32]—this work collectively demonstrates that the technical framework established in this era maintained sustained productivity over the following decade.

However, ZFP technology faced serious limitations: context-dependent effects between adjacent modules rendered design unpredictable [33]; requirements for specific recognition sequences restricted the range of targetable sites [34]; fused methyltransferases exhibited significant off-target activity [35]. Although these studies established the conceptual foundation, technical barriers impeded widespread adoption. Crucially, however, the narrative that ZFPs were simply superseded by superior platforms requires direct qualification. Cappelluti et al.‘s landmark study published in Nature (2024) conducted a head-to-head dose–response comparison of dCas9-, TALE-, and ZFP-based engineered transcriptional repressors (ETRs) targeting the *Pcsk9* locus in hepatocytes, finding that ZFP-ETRs were 5.7-fold more potent than dCas9-ETRs and 2.8-fold more potent than TALE-ETRs (EC50: ZFP-ETR 0.08, TALE-ETR 0.21, dCas9-ETR 0.38) [10]. In vivo, a single LNP dose of ZFP-ETR achieved approximately 60% reduction in circulating *Pcsk9* levels maintained for 330 days in mice, with CpG methylation at the *Pcsk9* promoter remaining stable through partial hepatectomy-induced liver regeneration [10]. The Neumann et al. CHARM study published in Science (2024) further illustrates the practical advantages of ZFPs: after initially optimizing the CHARM effector using the dCas9 platform, the authors deliberately replaced dCas9 with ZFPs for all brainwide in vivo experiments, explicitly citing two reasons—their compact size (roughly an order of magnitude smaller than SpCas9, enabling AAV packaging) and lower immunogenicity due to the absence of bacterial epitopes, in contrast to Cas9 which is likely to become antigenic upon chronic AAV-driven expression [14]. Together, these data demonstrate that ZFPs can outperform dCas9-based platforms in potency, and offer distinct advantages in delivery-constrained (single-AAV) or immunosensitive (CNS, repeated-dosing) contexts—a comparative reality the field should acknowledge honestly.

### Second-generation systems: the transitional breakthrough of TALEs (2009–2013)

In October 2009, Boch et al. and Moscou and Bogdanove independently published in Science the deciphered DNA-binding code of transcription activator-like effectors (TALEs) [36, 37]. TALEs derived from Xanthomonas follow a simple ‘one-to-one’ recognition rule: each 33–35-amino-acid repeat unit recognizes a single nucleotide through the repeat variable di-residue (RVD) at positions 12 and 13 (NI→A, HD→C, NG→T, NN→G) [38, 39]. Unlike ZFPs, TALE modules are functionally independent, with no context interference, reducing construction time from weeks to days [40].

The year 2013 was pivotal for epigenetic editing: Joung’s group developed the TALE-TET1 system for targeted demethylation at specific loci (including KLF4 and HBB), demonstrating that modification of methylated promoter CpGs could activate endogenous gene expression [41]; Bernstein’s group created TALE-LSD1 for removing H3K4me1/me2 from enhancers, establishing ‘epigenetic knockdown’ as a paradigm for functional studies [42]; Zhang Feng’s group developed the light-controlled TALE system LITE for inducible epigenetic regulation [43].

Despite these advances, TALEs continued to face persistent challenges: limited binding to methylated DNA and large protein sizes complicated delivery [44]. More critically, the rise of CRISPR-Cas9 in 2012 rapidly overshadowed TALE research—retargeting required replacing approximately 20 nucleotides of guide RNA rather than redesigning the entire protein [45].

### Third-generation systems: the CRISPR/dCas9 revolution (2013–present)

#### Core advantages of the dCas9 platform relative to Cas9

In early 2013, Lei Stanley Qi and colleagues at UCSF, together with the laboratories of Jennifer Doudna, Jonathan Weissman, Adam Arkin, and Wendell Lim, published in Cell a study that fundamentally altered the trajectory of epigenetic editing: by inactivating Cas9 nuclease activity through two point mutations (D10A and H840A), they created dCas9 (catalytically dead Cas9)—a variant that retains DNA-binding capability but is unable to cleave DNA [46]. Later that year, Gilbert, Qi, Weissman, and colleagues extended CRISPRi to mammalian cells by fusing dCas9 to a KRAB repressor domain, achieving robust transcriptional silencing [12]; simultaneously, three independent groups—including Gilbert et al. [12], Perez-Pinera and Gersbach et al. [47], and Maeder and Joung et al. [48]—demonstrated that dCas9 fused to transcriptional activation domains (VP64 or p65AD) could activate endogenous gene expression in human cells, collectively establishing dCas9 as a modular platform for programmable transcriptional regulation. Compared with Cas9, the core advantages of the dCas9 platform include: no production of DNA double-strand breaks, fundamentally circumventing the risk of off-target mutations and chromosomal rearrangements; modular design allowing fusion to diverse effector domains; retargeting to any genomic position requiring only sgRNA exchange; and maintenance of reversibility of epigenetic modifications [46, 49].

#### Iterative evolution of effector domains

Between 2015 and 2017, epigenetic editing tools underwent explosive expansion. In 2015, Charles Gersbach’s group at Duke University demonstrated for the first time that direct editing of histone acetylation can activate genes from promoters and enhancers by fusing dCas9 with the catalytic core of human p300 acetyltransferase (dCas9-p300) [50]; the same year, a highly specific dCas9-KRAB repression system was developed for silencing of distal regulatory elements [51]. In 2016, targeted DNA methylation editing underwent a breakthrough as multiple independent groups near-simultaneously reported dCas9-DNMT3A fusion systems in mammalian cells. Among these, Vojta et al. were the earliest to publish, demonstrating heritable CpG methylation at the IL6ST and BACH2 loci [52], while McDonald and Challen et al. independently reported site-specific methylation of tumor suppressor gene promoters [53]. The Jaenisch laboratory at the MIT Whitehead Institute provided the most functionally comprehensive study, simultaneously demonstrating dCas9-DNMT3A for targeted de novo methylation and dCas9-TET1 for targeted demethylation, with applications ranging from gene silencing to chromatin loop remodeling and in vivo validation in mice [54]. In 2017, Stepper et al. further improved methylation efficiency by 4–5-fold through fusion of DNMT3A with its cofactor DNMT3L (dCas9-DNMT3A-3 L) [55].

However, first-generation CRISPRi/CRISPRa systems harbored a fundamental limitation: their effects depended on continuous dCas9 expression and typically disappeared within 3–7 days following transient transfection [12, 48, 53]. Even optimized dCas9-DNMT3A systems exhibited gradual loss of silencing within 2–4 weeks due to active demethylation by TET enzymes [56].

The CRISPRoff system published in Cell in 2021 represented a major leap in epigenetic editing. Developed by Nuñez et al. from the Gilbert and Weissman laboratories, the system fuses dCas9 with a tripartite effector containing KRAB, DNMT3A, and DNMT3L domains [11]. Transient expression establishes durable, heritable gene silencing maintainable across more than 450 cell division cycles. The companion CRISPRon system achieves reversal of silencing through fusion of TET1 catalytic domain with dCas9, combined with modified sgRNAs carrying MS2 stem loops to recruit trans-activation domains [11]. The molecular durability of CRISPRoff is rooted in the synergistic co-deposition of dual epigenetic marks: KRAB initiates H3K9me3 deposition by recruiting the KAP1-SETDB1 complex, while DNMT3A-DNMT3L establishes DNA methylation [11, 57]. These two marks form a self-reinforcing circuit: H3K9me3 recruits additional DNA methyltransferases through PWWP domain interactions, while DNA methylation recruits H3K9 methyltransferases through methyl-CpG binding proteins [58–60]. This mutual reinforcement creates a bistable epigenetic state resistant to cellular removal mechanisms.

The CHARM system published in Science in 2024 introduced a fundamentally different design philosophy to address delivery limitations [14]. Rather than expressing exogenous methyltransferases, CHARM recruits and activates endogenous DNMT3A through a compact fusion protein comprising a zinc finger DNA-binding domain, the C-terminal domain of DNMT3L, and an unmethylated histone H3 N-terminal tail. This ‘no exogenous methyltransferase’ design is sufficiently compact to be packaged within a single AAV vector, overcoming the bottleneck of CRISPRoff being too large for AAV delivery. Through AAV-PHP.eB delivery, CHARM achieved greater than 80% whole-brain knockdown of the prion protein gene in mice, with kinetically tuned self-silencing properties limiting chronic transgene expression [14].

#### From tool to therapy: technical foundations of clinical translation

Epigenetic editing is transitioning from laboratory to clinic. The most representative clinical advances currently include two landmark programs: EPI-321, developed by Epicrispr Biotechnologies (co-founded by Stanley Qi), targets the D4Z4 region in facioscapulohumeral muscular dystrophy to silence the pathogenic DUX4 gene by restoring methylation [16]—the first patient was dosed in August 2025, and the program has received FDA Fast Track, Rare Pediatric Disease, and Orphan Drug designations. TUNE-401, developed by Tune Therapeutics (co-founded by Charles Gersbach), targets chronic hepatitis B to permanently silence the HBV genome through epigenetic editing [15]—clinical trials were approved in New Zealand in November 2024 and in Hong Kong in January 2025, with preclinical data demonstrating viral silencing maintainable for more than 550 days and 275 cell doublings.

Contemporary epigenetic editing systems satisfy the fundamental requirements for therapeutic application through three characteristics. First, durability fundamentally addresses the challenge of repeated dosing—silencing established by a single dose in mice approaching one year, maintaining stable maintenance through liver regeneration induced by partial hepatectomy (involving substantial cell division), and reducing circulating PCSK9 levels by approximately 90% in non-human primates, transforming the treatment paradigm from chronic therapy to potentially one-time intervention [9, 10]. Second, reversibility provides a fundamental safety mechanism (detailed in Section Multi-layered safety mechanisms of epigenetic editing) [9]. Third, compact system designs such as CHARM overcome the delivery bottlenecks that initially constrained the field, enabling single-AAV delivery for neurological applications [14].

### Molecular determinants of epigenetic memory stability: quantitative kinetics and tissue context

The combination properties of the editor, effector type, and target cell collectively determine the fate of epigenetic silencing—a core issue that has received far less attention in existing reviews than it merits. This section deepens mechanistic understanding through three interrelated questions.

#### Quantitative relationship between methylation density and stability

The quantitative requirements for stable epigenetic silencing remain in an early stage of investigation, with current understanding derived primarily from experimental observation rather than rigorous kinetic modeling. In human induced pluripotent stem cells and embryonic stem cells, stable long-term silencing by CRISPRoff correlates closely with CpG methylation density at the target promoter: studies indicate that methylation of more than 70% of CpG sites within a ± 500 bp window around the transcription start site is associated with durable repression across hundreds of cell divisions [11]. When methylation density falls below approximately 40–50%, active demethylation by TET enzymes can overcome DNMT1-mediated maintenance methylation, leading to gradual reversal of silencing within 2–4 weeks [56]. These values represent a distillation of existing experimental data rather than independently validated kinetic models—the mutually reinforcing relationship between H3K9me3 and DNA methylation has theoretical bistable characteristics [58–60], but the precise threshold methylation density has not been quantified through direct kinetic experiments and should be regarded as qualitative guidance rather than a strict quantitative threshold.

The spatial distribution of methylated CpGs is equally important as overall density: methylation concentrated at CpG islands overlapping core promoter elements (particularly TATA box-proximal and Sp1 binding site regions) provides greater stability than equivalent density of peripheral CpG methylation. This principle has been directly validated in functional studies of primary human T cells—Goudy et al.‘s all-RNA CRISPRoff/CRISPRon platform demonstrated that epigenetic silencing targeting core promoter CpG islands remains stably maintained through multiple cell divisions, repeated T cell activation, and in vivo adoptive transfer [61]. Additionally, synthetic biology studies constructing CpG density gradient promoter libraries confirmed that single CpG sites between the TATA box and transcription start site exert disproportionate deterministic effects on silencing kinetics, further supporting the functional priority of core promoter CpG spatial positioning [62]. These thresholds are not universally applicable—they are modulated by chromatin context (promoter, gene body, or enhancer), cell type-specific TET and DNMT1 expression levels, and co-regulatory factors such as HP1α/β [58–60].

#### Differential analysis of CHARM in the brain versus CRISPRoff in the liver

The starkly different design requirements for CHARM (brain) and CRISPRoff (liver) are not coincidental but reflect fundamental differences in cell biology with direct implications for therapeutic design. In post-mitotic neurons—the primary target cells of CHARM—DNA replication is absent under normal conditions, eliminating the primary mechanism of methylation dilution. Once endogenous DNMT3A activation mediated by the H3 tail decoy deposits de novo CpG methylation at the Prnp locus, the primary threat to stability is TET-mediated active demethylation. The relatively constrained TET1/2/3 activity in terminally differentiated neurons under homeostatic baseline conditions—combined with chromatin compaction from co-deposited H3K9me3—limits active demethylation’s ability to eliminate newly established methylation, allowing silencing established by transient CHARM expression to persist [14].

In contrast, hepatocytes present an entirely different context. Although adult hepatocytes are primarily quiescent (typical turnover approximately 200–300 days), partial hepatectomy, chemical injury, or the high metabolic activity of the liver can drive substantial cell proliferation [63]. The persistence of ZFP-ETR silencing through 70% hepatectomy-induced regeneration—a process completing 2–3 rounds of cell division within 5–7 days post-surgery—directly demonstrates that the complete KRAB-DNMT3A-DNMT3L tripartite effector can establish sufficient methylation density and H3K9me3 co-deposition for faithful replication-coupled maintenance [10]. CHARM relies on endogenous DNMT3A levels, which vary by tissue and may be insufficient in hepatocytes or highly proliferative contexts, most likely explaining why CHARM has not yet been validated in liver applications. This analysis yields a design principle: compact editors recruiting endogenous machinery (CHARM-type) are best suited for post-mitotic, low-TET-activity tissues; complete tripartite effectors (CRISPRoff-type) are necessary for mitotically competent or high-TET-activity tissues.

#### The demethylation threat in non-dividing cells

In non-dividing cells, replication-dependent methylation dilution is absent, but active demethylation driven by TET-mediated oxidation is the primary threat to long-term stability. TET1-3 enzymes sequentially oxidize 5-methylcytosine (5mC) to 5-hydroxymethylcytosine (5hmC), 5-formylcytosine (5fC), and 5-carboxylcytosine (5caC), the latter two being substrates for thymine-DNA glycosylase (TDG)-mediated base excision repair, ultimately regenerating unmodified cytosine [56]. However, neuronal TET activity is not silent: despite relatively constrained baseline levels, this pathway remains dynamically regulated as a component of synaptic plasticity and cognitive function—TET1 knockout mice exhibit learning and memory deficits, indicating that functional TET activity exists in neurons and can be upregulated during neuronal activation, oxidative stress, and aging [64]. This dynamic regulatory characteristic, rather than simple ‘low activity,’ is key to accurately understanding CHARM’s long-term stability boundaries.

That CHARM-mediated silencing is maintained in mouse neurons despite the presence of active demethylation mechanisms suggests that H3K9me3 co-deposition reduces TET enzyme accessibility to methylated CpGs through HP1-mediated chromatin compaction—essentially using higher-order chromatin structure as a protective barrier. However, this protection may depend on cell state: neuronal activation, oxidative stress, and aging are all associated with increased TET activity and 5hmC accumulation. More broadly, for non-neuronal, non-dividing cells with appreciable TET activity—including cardiomyocytes (relevant to cardiac channelopathies) and skeletal muscle fibers (relevant to FSHD)—whether current epigenetic editors can achieve stable long-term silencing remains an open question of substantial clinical significance. Whether CHARM-mediated silencing can resist decades of TET activity in aging human neurons—particularly in the context of neurodegenerative disease with elevated oxidative stress—has not been validated and cannot be inferred from existing murine data. The comparative characteristics of these four generations of DNA-binding protein platforms are summarized in Table 1.

**Table 1:** Comparative analysis of DNA-binding protein platforms for epigenetic editing

Comparison	ZFP (1st Gen)	TALE (2nd Gen)	dCas9 (3rd Gen)	De novo DBP
Discovery Year	1985	2009	2013	2025
Protein Size	~ 30aa/finger	~ 34aa/repeat	~ 1368aa	< 65aa
Coding Sequence	~ 0.5-1 kb (6-finger array)	~ 1.5-2.5 kb (15–25 repeats)	~ 4.1 kb	< 200 bp
Recognition Mode	3 bp/finger	1 bp/repeat	20 bp sgRNA	6 bp
PAM Requirement	None	5’-T preference	NGG (SpCas9)	None
Design Timeline	Weeks	Days	Hours	Computational
AAV Delivery	Possible	Difficult	Difficult	Easy

## AI-driven precision design and optimization: achievements, limitations, and translational gaps

The convergence of artificial intelligence and epigenetic editing represents a fundamental shift from empirical optimization to computationally driven design. Before systematically reviewing specific AI tools, however, an objective assessment of their current translational status is a necessary prerequisite for maintaining scientific rigor. The most clinically advanced epigenetic editing programs were all designed using conventional molecular biology methods, with an evident temporal gap from the recent AI breakthroughs. While fully acknowledging these genuine contributions, this section will also provide a systematic assessment of the limitations of each class of AI tools, to avoid conflating ‘potential’ with ‘realized achievements.’

### From structure prediction to de novo design: evolution of the AI protein design toolchain

The reshaping of epigenetic editing design paradigms by AI began with the release of AlphaFold2 in July 2021. Jumper et al. reported in Nature that this system achieved median backbone RMSD of 0.96 Å in the CASP14 competition, with approximately two-thirds of targets scoring above 90 GDT, for the first time bringing protein structure prediction to experimental-grade accuracy [65]. This breakthrough earned Demis Hassabis and John Jumper a share of the 2024 Nobel Prize in Chemistry, and established a foundation for structural validation of subsequent de novo protein design work. In 2024, Abramson et al.‘s AlphaFold3 extended prediction scope to biomolecules including protein–nucleic acid complexes, adopting a new diffusion architecture to replace the original structure module [66]. For protein–nucleic acid complex prediction—including protein–DNA interactions of direct relevance to epigenetic editor design—AlphaFold3 demonstrates substantially higher accuracy than RoseTTAFold2NA [66]. For protein–ligand interactions, AlphaFold3 achieves a 76% success rate on the PoseBusters benchmark compared with 24% for AutoDock Vina, representing far greater accuracy than state-of-the-art docking tools [66]. Together, these advances are of direct relevance to epigenetic editing: AlphaFold3 can predict the complex structure of designed DNA-binding proteins with their target DNA sequences, providing reliable computational validation for rational design of targeting modules.

At the sequence design level, Dauparas et al.‘s ProteinMPNN, published in Science in 2022, pioneered message-passing neural network-based sequence design [67], achieving 52.4% native sequence recovery compared with 32.9% for conventional Rosetta methods, with approximately 200-fold improvement in computational speed. In 2023, Watson et al. introduced diffusion models into protein design through RFdiffusion [68], achieving experimental success rates approximately two orders of magnitude higher than previous methods and successfully designing protein binders with picomolar affinity. In the same year, Ichikawa et al.‘s work published in Nature Biotechnology (2023) established the first general deep learning model specifically for DNA-binding proteins, systematically optimizing zinc finger protein (ZFP) specificity for target DNA sequences and achieving transcription factor reprogramming in cellular experiments [69]. The model, by integrating large-scale ZFP-DNA binding data, substantively overcame the context-dependent effects at the sequence-specificity prediction level that had long plagued modular design in first-generation platforms—namely, the non-independence between adjacent zinc finger modules that rendered design unpredictable, a core bottleneck of the first-generation platform (see Section First-generation systems: the zinc finger protein era (1997–2009)). In terms of AI contribution tiers, this work belongs to Tier 2: it does not generate entirely new scaffolds but advances the performance boundaries of existing platforms toward computational predictability; no clinical program has yet incorporated AI-optimized ZFPs as core targeting modules, but this work indicates that AI-assisted ZFP design possesses the potential to be reactivated and laid a methodological foundation for subsequent de novo DNA-binding protein design. In 2024, Krishna et al.‘s RoseTTAFold2NA All-Atom, published in Science, further extended design capabilities to DNA, RNA, small molecules, metal ions, and covalent modifications [70], directly applicable to design and modeling of protein–DNA interactions in epigenetic editing.

In 2025, Glasscock, Pecoraro, McHugh, Baker, and colleagues published landmark work on de novo design of programmable DNA-binding proteins [17]. The research team developed a complete computational design pipeline: first generating approximately 26,000 helix-turn-helix scaffolds (fewer than 65 amino acids) from metagenomic sequences, followed by protein–DNA docking using an extended RIFdock approach, sequence design with Rosetta or LigandMPNN [71], and final screening validation with AlphaFold2. This method generated binders with 30–500 nM binding affinity for 5 different DNA targets, with single binding modules achieving sequence-specific recognition at up to 6 bp positions. Crystal structure validation revealed a Cα-RMSD of only 0.64 Å between the designed protein DBP-48 and its target DNA complex, in excellent agreement with the design model. In functional validation, these designed proteins achieved up to 20-fold transcriptional repression in E. coli, and 3–5-fold transcriptional activation in HEK293T cells upon fusion with VP64.

**Fig. 2:**
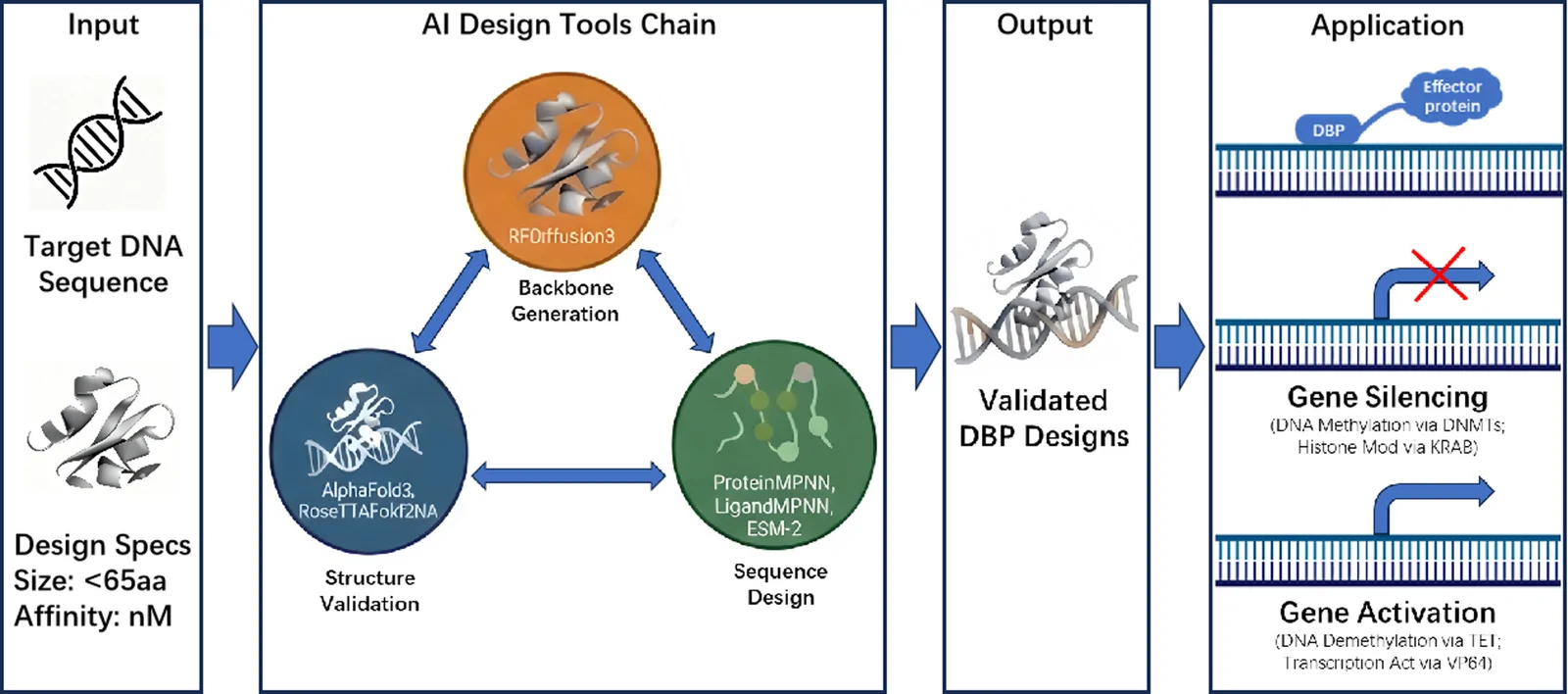
Integrated AI workflow for de novo DNA-binding protein design in epigenetic editing applications. The computational pipeline comprises three interconnected modules: backbone generation via diffusion models (RFdiffusion), sequence optimization using message-passing neural networks (ProteinMPNN/LigandMPNN/ESM-2), and structure validation through deep learning predictors (AlphaFold3, RoseTTAFold2NA). These tools operate in an iterative design-test cycle, enabling rational design of compact DNA-binding proteins (< 65 amino acids) with nanomolar affinity for subsequent fusion with epigenetic effector domains. Key performance metrics are indicated for each tool. The 2025 milestone achievement of programmable DBP design represents a paradigm shift from CRISPR-dependent targeting to fully de novo designed targeting modules. DBP: DNA-binding protein

Compared with TALEs (coding sequences exceeding 2000 bp) and dCas9 (SpCas9 approximately 1368 amino acids), these miniature DNA-binding proteins (50–65 amino acids) offer tremendous delivery advantages, readily fitting within a single AAV vector. More importantly, de novo designed DNA-binding proteins do not require PAM sequences, theoretically targeting any genomic locus, transcending the inherent limitations of CRISPR systems. The paper explicitly notes that these designed proteins can be linked to ‘epigenetic modifiers or other effector recruitment domains,’ providing novel targeting module options for next-generation epigenetic editing tools. Reviewing the evolutionary trajectory of this toolchain—from AlphaFold2’s structure prediction (2021), ProteinMPNN’s sequence design (2022), the parallel advancement of RFdiffusion and ZFP deep learning optimization (2023), RoseTTAFold2NA All-Atom’s multi-molecular extension (2024), to experimental validation of de novo DNA-binding proteins (2025)—reveals clearly that AI contributions to epigenetic editing targeting module design follow a progressive logic of ‘prediction → optimization of existing scaffolds → de novo generation,’ with each step building on its predecessor, and currently in the critical transition from proof-of-concept to clinical translation in Fig. 2.

### AI engineering optimization of epigenetic modifying enzymes and miniature editors

Protein language models provide powerful sequence–function prediction capabilities for enzyme engineering. ESM-2, published in Science in 2023 by Lin et al. and trained on protein sequences from the UniRef database [72], achieves a Spearman correlation of 0.415 in zero-shot mutational effect prediction and can predict three-dimensional structure from a single sequence. An automated evolution platform based on ESM-2 achieved a 2.4-fold improvement in tRNA synthetase activity within 10 days. ProGen, published in Nature Biotechnology in 2023 by Madani et al. and trained on 280 million protein sequences across more than 19,000 protein families [73], is a 1.2-billion-parameter autoregressive protein generation model; artificially generated lysozymes retain catalytic efficiency comparable to natural lysozymes even at sequence identity as low as 31.4%, with 73% of ProGen-generated proteins exhibiting functional activity.

AI-guided miniature editor engineering is key to addressing the AAV delivery bottleneck. TEEP—TnpB ωRNA efficiency predictor—published in Nature Methods in 2024 by Marquart et al. [74], achieves independent validation r values exceeding 0.84 in HEK293T cells and 0.82 in Neuro-2a cells; TEEP prediction-guided TnpBmax achieved a 4.4-fold improvement in editing efficiency over wild-type, with in vivo editing efficiencies reaching 75.3% in mouse liver and 65.9% in mouse brain. IscB engineering by Xue et al., published in Molecular Cell in 2024, through structure analysis-guided rational design, identified three arginine substitutions achieving an average 7.5-fold activity improvement [75], with the eIscB-D variant combined with HMG-D fusion achieving a peak editing efficiency of 91.3%.

It is noteworthy that the Fanzor [20] and IscB [21, 75] engineering achievements described above are fundamentally gene-editing tools with nuclease activity, with their relevance to epigenetic editing currently manifested primarily in exploratory work on fusing catalytically inactive versions with epigenetic effector domains. Fanzor engineering by Wei et al., published in Nature Chemical Biology in 2026, used AlphaFold3 to predict the NlovFz2 nuclease-ωRNA complex structure, guiding engineering to achieve an 11.1-fold improvement in gene editing efficiency over wild-type [20], with restoration of dystrophin expression in humanized Duchenne muscular dystrophy mouse models. The NovaIscB system, published in Nature Biotechnology in 2026, achieved approximately 100-fold activity improvement through evolution-guided protein design, reaching 40% indel activity [21], and integrated IscB with epigenetic modification domains to achieve durable in vivo epigenomic editing, providing a new direction for miniature epigenetic editor development—this is currently the closest exploration to directly applying small CRISPR proteins to epigenetic editing, though still at considerable distance from clinical application.

#### Three-tier integrated assessment of AI contributions

A comprehensive assessment of AI contributions in the epigenetic editing field clearly distinguishes the three tiers described in Section Introduction: accelerated LNP formulation screening has played a practical role in programs such as TUNE-401 (Tier 1); AlphaFold3-guided Fanzor engineering and NovaIscB applications in epigenetic editing remain at the cellular validation stage (Tier 2); de novo designed DNA-binding proteins in 2025 represent a proof-of-concept milestone, with no program incorporating them as core targeting modules to date (Tier 3). The following Section AI-enabled intelligent delivery systems focuses on evaluating the translational delivery value of Tier 1 contributions and their limitations.

### Current challenges and improvement opportunities for AI methods

While fully acknowledging the significant advances achieved by AI tools, understanding their current performance boundaries is equally essential for guiding future development. Regarding RFdiffusion, its experimental success rate is approximately 19%—meaning approximately one in five designs is experimentally validated as successful—a 2025 bioRxiv preprint (not yet peer-reviewed) evaluating its design performance across 6 targets encountered low expression, non-specific binding, or undetectable affinity in 5 eukaryotic targets [76]. A 2024 review summarized the experimental success rate of current state-of-the-art design approaches as close to 20%, indicating significant room for methodological improvement [77]. Regarding AGILE, the reported correlation coefficient of *r* = 0.78 between in vitro HeLa cell transfection and intramuscular delivery performance means approximately 40% of variance remains unexplained; this 7.8-fold improvement was measured in mouse muscle, and species differences (mouse → primate) further limit direct translational value; to date, no LNPs designed by AGILE have entered clinical trials [18].

Regarding COMET, its reported Spearman correlation of approximately 0.9 requires interpretation in methodological context: this correlation was obtained through cross-validation within the training set, not tested on an independent external dataset; whether its predictive capability generalizes to entirely novel lipid chemistry spaces (particularly brain-targeting lipids significantly divergent from the training data distribution) has not been validated; to date no COMET-designed formulations have entered clinical trials, and this tool is most accurately described as ‘accelerating formulation optimization within known chemical space’ rather than ‘designing entirely novel therapeutic delivery systems’ [19]. Regarding sgRNA prediction models, one study found that when five prediction algorithms were tested on an independent dataset of 430 sgRNAs, the correlation between predicted and measured activity was only *r* = 0.04 to 0.20 [78], indicating significant limitations in cross-dataset generalization. Even in replicate experiments, Spearman correlations between different cell lines ranged only from 0.531 to 0.752, suggesting that a correlation of approximately 0.7 may represent the practical upper limit at current experimental reproducibility.

Regarding AlphaFold2/3 for mutational effect prediction, differences between wild-type and mutant structure predictions typically remain below 1 Å Cα RMSD even when functional changes are substantial [79]; correlations between pLDDT scores and mutation-induced stability changes are weak [80], reflecting the intrinsic limitations of current static structure prediction methods. Additionally, membrane proteins constitute less than 3% of PDB structures, causing AlphaFold2 to be biased toward soluble proteins in its training [80]. The challenges in sgRNA prediction have deeper underlying causes: training data generated by different laboratories, cell lines, and assay methods contain systematic variation, making it difficult for models to distill universally applicable rules. The poor interpretability of deep learning models—their ‘black box’ nature—makes it difficult to derive biological mechanisms from predictions [81]. Robust validation strategies—including leave-one-sgRNA-out cross-validation, leave-one-study-out cross-validation to test cross-platform generalization, and independent dataset validation—are essential for ensuring actual predictive capability of models [82]. The principal AI tools discussed in this section and their applications in epigenetic editing are summarized in Table 2.

**Table 2:** Summary of key AI tools for epigenetic editing design and optimization

Tool Name	Year	Primary Function	Application
AlphaFold2	2021	Protein structure prediction	Design validation
AlphaFold3	2024	Protein-DNA/RNA/ligand complex prediction	Editor-DNA interaction prediction
ProteinMPNN	2022	Protein sequence design	Effector domain optimization
LigandMPNN	2025	Ligand-aware sequence design	DNA-binding interface optimization
RFdiffusion	2023	De novo protein backbone design	Novel scaffold design
ESM-2	2023	Protein language model	Mutation effect prediction
DBP Design	2025	De novo DNA-binding protein design	Next-gen targeting modules

#### AI prediction of targeting module immunogenicity

A prospective direction. The discussion of AI tool limitations above reveals a direction that has not received adequate attention: immunogenicity prediction. Targeting modules of epigenetic editors—whether dCas9, zinc finger proteins, or de novo designed DNA-binding proteins—are all foreign proteins in the human body and can induce immune responses affecting durability of efficacy and limiting the feasibility of repeated dosing. Importantly, immunogenicity concerns extend beyond targeting modules to the epigenetic effector domains themselves—KRAB, DNMT3A, DNMT3L, and TET1 catalytic domains are all potentially immunogenic proteins whose expression duration and re-exposure risk depend directly on delivery strategy and clinical scenario [83]. For hit-and-run LNP-mRNA delivery, transient expression limits but does not eliminate immune priming; for AAV-delivered systems with prolonged transgene expression, sustained effector protein presentation to the immune system poses a more substantial risk. In re-dosing scenarios—whether due to insufficient initial silencing or administration of activating editors for reversal—patients face repeated exposure to the same effector proteins, compounding immune sensitization risk. AI-driven redesign of effector domains to reduce immunogenicity or enhance human compatibility therefore represents an important but currently underexplored avenue for improving safety: epitope prediction tools such as NetMHCpan [84] and DeepHLApan [85] can systematically identify high-risk MHC-I/II binding regions within effector sequences, while sequence design tools such as ProteinMPNN can in principle mutate these regions while preserving catalytic function—an approach established in antibody humanization and enzyme replacement therapy but not yet systematically applied to epigenetic effector engineering to date. We foresee AI can play a role at two levels: First, epitope prediction—existing deep learning tools (such as NetMHCpan, DeepHLApan) can systematically scan protein sequences for MHC-I/II binding epitopes and can in principle identify high-immunogenicity regions in targeting modules during the design stage; Second, de-immunization design—combined with sequence design tools such as ProteinMPNN, high-risk epitopes can be mutated while retaining DNA-binding function to generate variants with reduced immunogenicity. This approach has been applied in antibody humanization and enzyme therapies but has not been systematically integrated into epigenetic editor design workflows to date. For clinical scenarios requiring activators to achieve reversibility, immunogenicity management is particularly critical—patients need to receive the same or similar protein months to years after initial dosing, and pre-existing immunity may significantly affect the safety and efficacy of activators. AI-assisted immunogenicity prediction and de-immunization engineering represent an important frontier for extending AI contributions from functional optimization to clinical safety design.

## AI-enabled intelligent delivery systems

The challenges of delivering epigenetic editors differ fundamentally from those of conventional gene therapy. This distinction stems from the unique pharmacological characteristics of epigenetic editing: transient editor expression can establish durable, heritable changes, obviating the need for continuous transgene activity while imposing stringent requirements on initial delivery efficiency and tissue specificity. The ‘hit-and-run’ paradigm—where the editor acts only transiently to deposit self-sustaining epigenetic marks—has catalyzed deep integration of delivery science and artificial intelligence.

### Special requirements and challenges for epigenetic editor delivery

This hit-and-run mechanism imposes delivery requirements that differ fundamentally from those of conventional gene therapy in two respects: whereas traditional CRISPR-Cas9 gene knockout tolerates sustained or repeated transgene expression to achieve cumulative editing, epigenetic editors must achieve pharmacodynamically sufficient target engagement within a single transient expression window—making initial delivery efficiency and tissue specificity non-negotiable rather than optimizable parameters. Nuñez et al.‘s CRISPRoff system, published in Cell in 2021, first demonstrated that transiently expressed epigenetic editors can establish stable gene silencing persisting across hundreds of cell divisions in human cells [11], establishing the theoretical basis for the ‘hit-and-run’ delivery strategy. The landmark study by Cappelluti et al., published in Nature in 2024, advanced this concept to in vivo validation [10]: the research team used lipid nanoparticles to deliver mRNA encoding the ZFP-ETR epigenetic editor, demonstrating that silencing effects remained stably maintained through liver regeneration induced by partial hepatectomy—a process involving substantial cell division—confirming the central role of DNA methylation as the carrier of ‘epigenetic memory.’

However, delivery of epigenetic editors faces unique challenges. Size is the foremost issue: the complete CRISPRoff system coding sequence is approximately 6.2 kb, exceeding the packaging capacity of a single AAV vector when combined with sgRNA expression cassettes [86]. Tissue specificity constitutes a second barrier: conventional lipid nanoparticles administered intravenously accumulate highly in the liver [87, 88], advantageous for liver-targeting applications (such as PCSK9 silencing for cholesterol reduction) but a delivery barrier for non-hepatic targets such as the nervous system, muscle, or tumors. Immunogenicity is a third consideration: repeated dosing may induce anti-vector immune responses; the durability of epigenetic editing theoretically requires only a single dose, which partially mitigates but cannot fully eliminate this issue—some patients may require re-dosing when silencing diminishes, and administering activators to achieve reversibility also constitutes additional exposure. Beyond anti-vector responses, immune recognition of the effector protein cargo itself—including KRAB, DNMT3A, and TET1 domains—represents an additional immunogenicity dimension that has received insufficient attention in current clinical program design and warrants prospective monitoring in ongoing Phase I trials.

### The AI revolution in lipid nanoparticle design

Artificial intelligence is fundamentally transforming the paradigm of lipid nanoparticle formulation development, converting traditional empirical trial-and-error into data-driven rational design. The AGILE platform reported by Xu et al. in Nature Communications in 2024 is a benchmark achievement in this field [18]: employing a graph neural network architecture, pre-trained through contrastive learning on more than 60,000 virtual lipid structures with MolCLR molecular representation extraction, achieving a Pearson correlation of 0.78 between in vitro HeLa cell transfection and in vivo intramuscular delivery performance, and 41% accuracy in identifying top-performing lipids. In vivo validation, the H9 lipid formulation discovered by AGILE demonstrated 7.8-fold higher mRNA delivery efficiency in mouse muscle compared with clinical standard MC3, while showing lower ALT and AST levels, indicating improved safety characteristics.

However, the translational significance of AGILE’s in vitro-to-in vivo correlation results must be interpreted with appropriate caution. A fundamental challenge not yet addressed by AI-driven LNP design is the weak correlation between in vitro transfection efficiency and in vivo delivery performance. A systematic comparison of 96 LNP formulations indicated that this in vitro-in vivo correlation can be as low as *r* = 0.23 to 0.47 depending on target organ [89], meaning AI models trained primarily on cell line data may be selecting for in vitro artifacts rather than true determinants of in vivo efficacy. The *r* = 0.78 reported by AGILE is a meaningful improvement but still leaves approximately 40% of variance unexplained. More critically, AGILE was trained and validated on a limited set of formulation parameters, without systematic incorporation of protein corona formation, endosomal escape kinetics, or immune cell uptake—all of which are key determinants of in vivo performance and are tissue- and patient-specific [90].

**Fig. 3:**
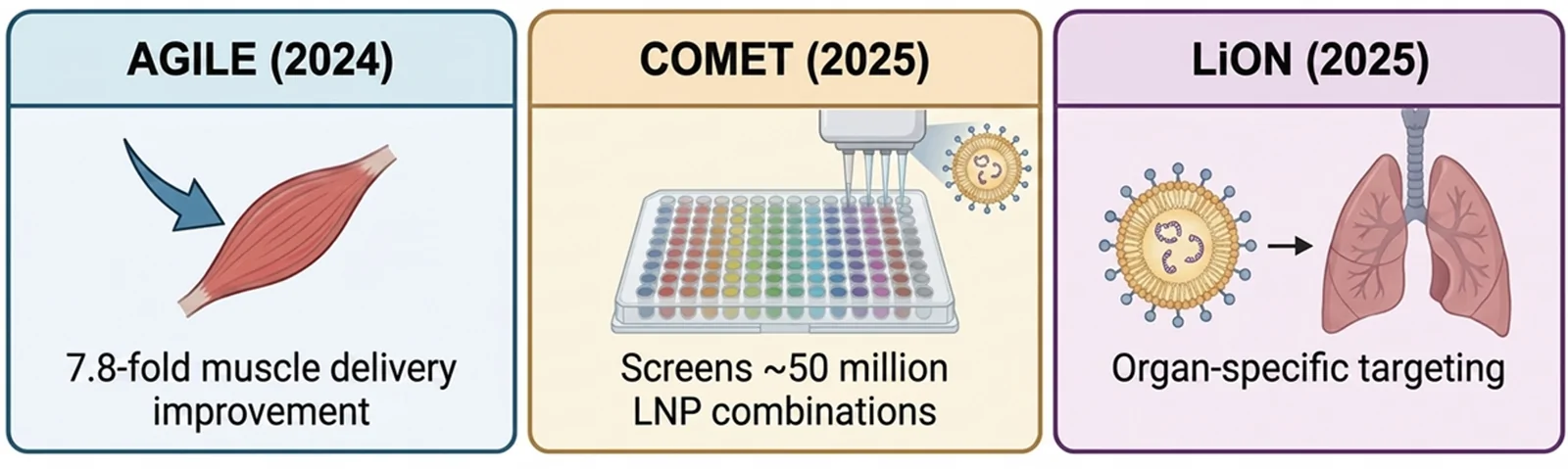
Artificial intelligence platforms for lipid nanoparticle optimization. Three approaches are compared: AGILE (AI-Guided Ionizable Lipid Engineering), COMET (Composite Material Transformer), and LiON (Lipid Nanoparticle Optimization Network). LNP: lipid nanoparticle; mRNA: messenger RNA

COMET, reported by Chan et al. in Nature Nanotechnology, introduced the Transformer architecture to LNP design, pioneering integrated modeling of multi-component LNP formulations as ‘tokens,’ using Uni-Mol for 3D molecular representation learning [19], trained on more than 3000 LNP formulation data points, capable of virtually screening approximately 50 million LNP combinations, with prediction performance Spearman correlation of approximately 0.9, and capable of predicting lyophilization stability. As noted above, this correlation was obtained from within-training-set cross-validation rather than an independent external dataset, and to date no COMET-designed formulations have entered clinical trials; its actual translational value remains to be prospectively validated. In late 2024, Witten et al.‘s LiON platform, published in Nature Biotechnology, focused on non-hepatic delivery scenarios [91], trained on more than 9000 LNP activity measurement data points, virtually evaluating 1.6 million lipids, and validated in mouse and ferret lung delivery models. Generative AI also shows potential for designing entirely novel ionizable lipids; a 2024 preprint reported a generative framework based on Synthesis-DAGs models achieving an 83.4% ionizable lipid generation rate [92], though experimental toxicity validation data for lipids designed by generative models remains very limited. A comparative overview of AI-driven LNP design platforms is shown in Fig. 3.

### Breakthroughs in tissue-specific delivery technology

Despite the significant improvement in LNP formulation optimization efficiency by AI platforms such as AGILE and COMET, the inherent high liver accumulation of conventional LNPs administered intravenously remains the core barrier for non-hepatic targeting applications. The SORT technology published by Cheng et al. in Nature Nanotechnology in 2020 systematically altered organ tropism by adding a fifth ‘SORT molecule’ to conventional four-component LNPs [93]: addition of 50 mol% cationic lipid DOTAP redirects delivery from liver to lung (associated with 108-fold vitronectin enrichment and αvβ3 integrin binding), while addition of 10–15% anionic lipid 18PA achieves spleen targeting (associated with 123-fold β-2-glycoprotein-1 enrichment). In 2026, Siegwart’s team reported dual SORT LNPs in Nature Biotechnology that simultaneously edited both hepatocytes and lung AT2 cells in an alpha-1-antitrypsin deficiency model, achieving simultaneous targeting of multiple organs [94].

**Fig. 4:**
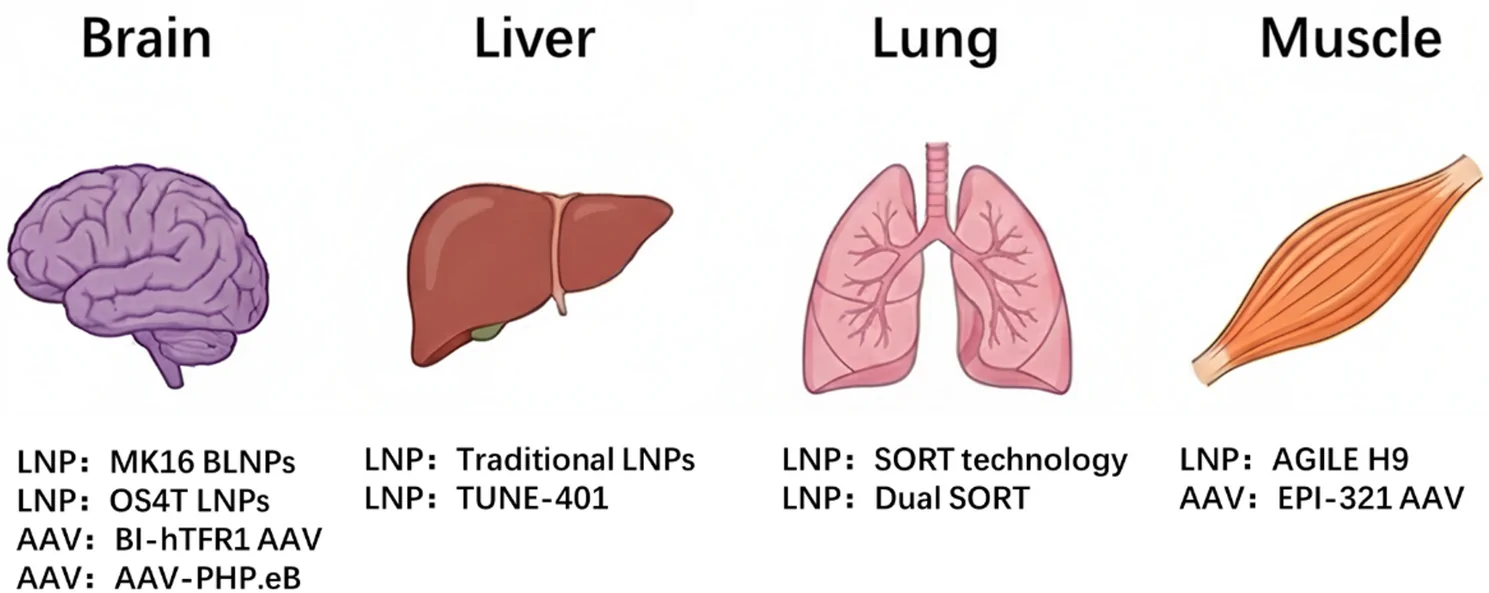
Tissue-specific delivery platforms and safety architecture for clinical epigenetic editing. Brain delivery includes MK16 BLNPs (8.3-fold over MC3) and OS4T LNPs (> 50-fold over Onpattro). Liver applications feature TUNE-401 for chronic HBV. Muscle targeting employs AGILE-optimized formulations and EPI-321 for FSHD. BLNPs: brain-penetrant lipid nanoparticles; AAV: adeno-associated virus; FSHD: facioscapulohumeral muscular dystrophy; HBV: hepatitis B virus

Crossing the blood-brain barrier is the core challenge for neurological epigenetic editing [95, 96]. In 2025, Wang et al. reported MK16 brain-penetrating LNPs in Nature Materials, selected from a library of 72 blood-brain barrier-crossing lipids; systemic injection achieved whole-brain transfection of neurons and astrocytes, with efficiency 8.3-fold higher than FDA-approved MC3, 7.4-fold higher than ALC-0315, and 6.5-fold higher than SM-102 [97]. Another study published in Science Advances developed OS4T LNPs integrating 5-HT3 receptor ligands, achieving more than 50-fold higher brain mRNA translation than Onpattro (MC3) in mice [98]. Transferrin receptor targeting strategies have also advanced; a 2025 Nature Nanotechnology study reported a universal antibody capture system using anti-Fc nanobody TP1107, achieving more than 1000-fold higher protein expression than non-targeted LNPs [99]. Tissue-specific delivery strategies optimized for different target organs are summarized in Fig. 4.

### AAV vector engineering and AI optimization

AAV vectors have unique advantages for epigenetic editor delivery, particularly suited for tissues where LNP access is limited and where sustained transgene expression is tolerable or can be mitigated by self-silencing designs such as CHARM—most typically the central nervous system, skeletal muscle, and cardiac muscle. Section  The AI revolution in lipid nanoparticle design has addressed LNP-based liver delivery; this section focuses on AAV engineering advances for the three major non-hepatic target tissues most relevant to clinical epigenetic editing programs.

CNS delivery. Deverman et al.‘s CREATE platform, published in Nature Biotechnology in 2016, inaugurated a new era of AAV capsid directed evolution [100], with the AAV-PHP.B capsid achieving 40–92-fold higher CNS transduction efficiency than AAV9. In 2020, the M-CREATE method further optimized results, with AAV-PHP.eB achieving equivalent neural transduction at 10-fold lower dose, representing up to 50-fold improvement over AAV9 [101]. Voyager Therapeutics’ TRACER platform uses RNA-based screening strategies enabling direct screening in non-human primates; the VCAP-102 capsid demonstrated 20–400-fold gene transfer improvement relative to AAV9 in multiple brain regions of both rodents and non-human primates [102]. The BI-hTFR1 system, published in Science in 2024, represents another milestone in whole-brain delivery [103], demonstrating 54-fold higher brain mRNA levels than AAV9 in humanized TFRC knock-in mouse models, with neuronal transduction rates of 71% and astrocyte transduction rates of 92%. Neumann et al. demonstrated that the CHARM system delivered via AAV-PHP.eB achieved more than 80% whole-brain PrP protein knockdown in mice [14], providing the most direct demonstration to date of CNS-targeted epigenetic editing. A critical translational caveat applies to all PHP-series capsids: their CNS tropism depends on the LY6A receptor, which is expressed in mouse brain vasculature but whose expression in human and NHP brain vasculature is insufficient to support PHP-series efficient blood-brain barrier crossing via the LY6A pathway [104, 105].

Skeletal muscle delivery. For muscle disease applications—most directly relevant to EPI-321’s treatment of FSHD—AAVrh74 has become the preferred clinical capsid. AAVrh74 efficiently transduces skeletal muscle via systemic or intramuscular administration and has established a favorable safety profile in multiple neuromuscular disease trials (including Duchenne muscular dystrophy programs). Its muscle tropism is maintained in both non-human primates and humans, providing a more direct translational pathway than PHP-series CNS capsids. However, the doses required for systemic AAVrh74 delivery to achieve whole-body muscle transduction raise the high-dose safety concerns described in Section Safety considerations and translational barriers for delivery systems, and the approximately 4.7 kb packaging capacity limits the size of epigenetic editor cargo that can be accommodated [86].

AI-accelerated capsid engineering. Machine learning is accelerating AAV capsid functional prediction across all tissue targets. Church laboratory’s study, published in Science in 2019, performed complete single codon substitution across all 735 positions of AAV2, generating a fitness landscape for approximately 200,000 variants [106]. In 2021, Bryant et al. in Nature Biotechnology analyzed 201,426 variants, finding more than 110,000 engineered capsids retaining activity, with deep neural networks capable of accurately predicting capsid activity with limited training data [107]. In 2024, Eid et al.‘s Fit4Function method, published in Nature Communications, achieved generalizable multi-trait AAV engineering, with 88% of library variants passing all 6 preset criteria, and top liver-targeting capsids demonstrating 10–1000-fold higher transduction efficiency of human hepatocytes than AAV9 [108]—indicating that AI-guided engineering can simultaneously optimize multiple functional attributes such as tissue tropism, production yield, and immune evasion.

### Development of emerging delivery platforms

Engineered virus-like particles integrate the advantages of LNPs and viral vectors, providing unique delivery options for epigenetic editors. Liu’s group’s fourth-generation eVLP system, published in Cell in 2022, packages base editing ribonucleoproteins at 16-fold higher efficiency than the initial design, and achieves 26-fold higher in vivo liver editing efficiency, with single injection achieving 63% liver editing and 78% serum *Pcsk9* reduction with nearly undetectable off-target editing [109]. In late 2024, fifth-generation eVLPs further improved delivery efficacy 2–4-fold through directed evolution [110]. In 2025, the RENDER platform specifically designed for the CRISPRoff epigenetic editor was published in Nature Communications [111], with the optimized v3 formulation achieving 99% silencing efficiency at the highest dose, representing a 4.4-fold efficacy improvement over the initial design, and successfully delivering to primary T cells and iPSC-derived neurons.

mRNA engineering optimization has significantly improved epigenetic editor expression efficiency. N1-methylpseudouridine modification has become the standard for mRNA drugs, providing 13–44-fold higher protein expression than pseudouridine-modified mRNA [112]. In 2025, Zhang et al.‘s GEMORNA platform, published in Science, using AI-driven codon optimization, achieved 41-fold higher luciferase expression and 15-fold higher mouse human EPO expression compared with conventional optimization methods [113]. Deep learning-guided 5’ UTR design has also achieved breakthroughs, with novel 5UTR05 combined with optimized 3’ UTR achieving more than 127% enhancement in translation efficiency [114]. Cell-penetrating peptides provide a carrier-free option for direct protein delivery; a 2024 study reported in Nature Communications demonstrated a self-deliverable Cas9 RNP system through A22p peptide improving editing efficiency by more than 3-fold [115].

### Safety considerations and translational barriers for delivery systems

Despite significant delivery technology advances, addressing safety considerations remains a core task for advancing clinical translation. The primary safety risks of lipid nanoparticles (intravenous route) include: hepatotoxicity (ALT/AST elevation) due to liver accumulation; pre-existing anti-PEG antibodies in 23–72% of healthy individuals (which can lead to accelerated blood clearance, reducing circulating half-life by 2–10-fold [116]); and complement activation-related pseudoallergic reactions (mRNA-LNP vaccines activate complement approximately 5-fold more than controls [117]).

High-dose AAV clinical trials have revealed important safety issues. A meta-analysis of 255 AAV clinical trials showed 11 patient deaths in 8 trials, with treatment-related serious adverse events in 30.6% of trials [118]. The ASPIRO trial (X-linked myotubular myopathy AT132) reported 3 patient deaths occurred in patients receiving the highest dose (3 × 10¹⁴ vg/kg) [119], all associated with progressive cholestatic liver disease and decompensated liver failure, and the FDA subsequently placed the trial on clinical hold. Epigenetic editing theoretically requires only a single dose, but initial delivery efficiency needs to be higher to compensate for editing efficiency losses; this requirement may drive toward higher doses, making safety margin issues more prominent.

Bridging the gap between AI predictions and in vivo effects is a key frontier of the field. Studies show that in vitro LNP delivery efficiency cannot reliably predict in vivo effects; certain formulations effective in vitro perform differently in vivo and vice versa [89]. Machine learning toxicity prediction models show variable performance, with some clinical toxicity models achieving AUC of 0.66–0.71, while advanced deep learning methods can reach 0.80–0.99, reflecting current limitations and improvement opportunities in AI safety prediction [120]. Species differences add complexity to extrapolation from animal models to humans; for example, AAV3B performs excellently in human hepatocytes but differently in mouse models [121].

### Mapping delivery technology maturity to clinical criteria fulfillment

Sections 2 through 4 have systematically described the technological maturation of epigenetic editing. Liver delivery has matured, skeletal muscle and whole brain are in early clinical validation stages, and evidence for post-mitotic tissues such as cardiomyocytes remains insufficient—this gradient directly determines the current degree to which Criterion 2 can be met in each target organ. Criterion 3 (initiation of human trials) depends on sufficient preclinical accumulation of the first two criteria but does not equate to human validation of Criteria 1 and 2. The following Section Clinical translation progress: a critical assessment using the three maturity criteria as a framework will systematically evaluate the evidence credibility of each clinical program within this logical framework. The current clinical development landscape of epigenetic editing therapies is summarized in Table 3.

**Table 3:** Current clinical development landscape of epigenetic editing therapies

Program	Company	Indication	Target	Delivery	Phase
OTX-2002	Omega Therapeutics	HCC & solid tumors	MYC	LNP	Phase I completed
TUNE-401	Tune Therapeutics	Chronic HBV	HBV cccDNA & intDNA	LNP	Phase I b
EPI-003	Epigenic Therapeutics	Chronic HBV	HBV cccDNA & intDNA	LNP	Phase I
CRMA-1001	nChroma Bio	Chronic HBV	HBV cccDNA & intDNA	LNP	Phase I/II
EPI-321	Epicrispr Biotechnologies	FSHD	DUX4 / D4Z4	AAV (AAVrh74)	Phase I/II
STX-1150	Scribe Therapeutics	Hypercholesterolemia	PCSK9	LNP	IND-enabling

## Clinical translation progress: a critical assessment using the three maturity criteria as a framework

Given the rapid pace of clinical development, some cited sources derive from company announcements and conference reports (not yet peer-reviewed). This section uses the three quantifiable maturity criteria established in the introduction as an analytical framework to systematically assess the degree to which current clinical and preclinical evidence supports each criterion.

Before proceeding to analysis of individual programs, it is necessary to explicitly link the delivery platform advances discussed in Section AI-enabled intelligent delivery systems to the specific delivery decisions of each program, as this mapping directly determines the evidence credibility of each program within the framework. TUNE-401 selects LNP/mRNA delivery, with its Criterion 2 evidence built upon the known limitations of LNP in vitro-in vivo correlation (*r* = 0.78, approximately 40% variance unexplained). EPI-321 selects AAVrh74, but must confront the high-dose AAV safety concerns (meta-analysis of 255 clinical trials showing treatment-related serious adverse events in 30.6%). CHARM’s AAV-PHP.eB is significantly less efficient in non-human primates than in mice, representing a key uncertainty for its clinical translation pathway. The following analysis will systematically evaluate each program within this framework.

For Criterion 1 (mechanistic durability), the strongest evidence comes from *Pcsk9* silencing maintained for more than 3 months in non-human primates and more than 12 months in humanized mice, and TUNE-401’s maintenance in continuously proliferating human cells for more than 550 days; for Criterion 2 (delivery competence), EPI-321’s systemic skeletal muscle transduction via AAVrh74 and CHARM’s more than 80% whole-brain coverage via AAV-PHP.eB provide evidence of tissue-selective delivery beyond the liver; for Criterion 3 (clinical validation), following the October 2022 first-in-human administration of OTX-2002 (MYCHELANGELO study), the initiation of human trials for TUNE-401 (from November 2024) and EPI-321 (August 2025) further expanded the clinical validation scope of this field, all with preset pharmacodynamic endpoints, representing important clinical advances in genetic and infectious disease indications. It must be candidly acknowledged that simultaneous satisfaction of all three criteria in humans has not yet been achieved—currently available clinical data comes only from Phase I early safety assessment stages, and human in vivo durability and delivery efficiency data remain to be accumulated.

### Criterion 1 validation: preclinical and early clinical evidence for mechanistic durability

#### TUNE-401 and chronic hepatitis B: the design logic for durable silencing

##### Technical basis and design rationale

TUNE-401 employs Tune Therapeutics’ proprietary TEMPO platform, delivered by lipid nanoparticles developed by Acuitas Therapeutics, with LNPs encapsulating mRNA encoding the epigenetic silencing protein and guide RNA. The TEMPO platform is a modular fusion protein comprising a modified non-cleavage DNA-binding domain with dual effector domains—a DNA methyltransferase and a transcriptional repressor domain. Tune Therapeutics has not publicly disclosed the specific type of DNA-binding domain (whether dCas9, zinc finger proteins, TALEs, or other scaffolds) nor the precise composition of effector domains, which remain proprietary information. The guide RNA targets a single conserved ‘master sequence’ shared between integrated viral DNA and extrachromosomal cccDNA across all known HBV genotypes, enabling a single construct to simultaneously silence both viral reservoirs. The mechanistic rationale lies in addressing the fundamental limitation of existing antiviral drugs: nucleoside analogs suppress viral replication but can neither eliminate the cccDNA template that persists in hepatocyte nuclei as chromatinized mini-chromosomes [122] nor silence integrated viral DNA—in chronically infected livers, integrated HBV DNA copy numbers exceed cccDNA by 10- to 1000-fold [123]. By directly targeting both forms of viral DNA with dual epigenetic marks, TUNE-401 aims to silence the transcriptional template rather than inhibit the replication machinery—if this effect is durable, it offers the prospect of functional cure rather than lifelong viral suppression. The LNP-mRNA delivery format is identical to the COVID-19 vaccine delivery paradigm and leverages the natural hepatic tropism of LNPs administered intravenously, requiring no additional tissue-targeting modifications to access the liver as the target organ.

##### Key preclinical evidence and its limitations

Preclinical data reported at the 2024 AASLD meeting showed up to 99.99% suppression of HBV RNA (from cccDNA) in primary human hepatocytes and humanized FRG mouse models, maintained for more than 550 days in continuously proliferating human cell lines. These data establish the concept of durable silencing across continuous cell divisions, directly confirming the epigenetic memory hypothesis in the HBV context, constituting strong support for Criterion 1.

However, three important limitations constrain direct inference of clinical success. First, the immunodeficient nature of the FRG mouse model poses constraints on its clinical predictive value far exceeding general animal model limitations. The Rag2/Il2rg double knockout in FRG mice eliminates T, B, and NK cells, completely abrogating the most critical effector arm in HBV infection pathology: HBV-specific CD8 + CTL-mediated hepatocyte killing. In chronic HBV patients, epigenetic silencing—even if it suppresses viral transcription—does not eliminate antigen expression from infected hepatocytes; persistent HBsAg and HBcAg presentation can still drive CTL-mediated immunopathology. In immunocompetent human patients, this persistent immune pressure may selectively clear silenced hepatocytes, thereby affecting treatment durability. To date, no study has evaluated TUNE-401’s durability in immunocompetent models (including non-human primates), representing the most significant gap in this program’s preclinical evidence chain. Second, durability data come from human cell lines maintained under artificial culture conditions, whose epigenomic landscapes differ substantially from chronically infected human liver. Third, TUNE-401 lacks published non-human primate efficacy data and quantitative cccDNA methylation endpoints.

##### Structural assessment of evidence adequacy

A comprehensive assessment of the predictive value of TUNE-401’s current preclinical evidence requires distinguishing three tiers of evidence reliability within the HBV biological context: cell line data (550-day durability) provides mechanistic proof-of-concept, but cell line epigenomic landscapes differ substantially from chronically infected liver, limiting predictive power; humanized FRG mouse data (up to 99.99% suppression) demonstrates functional activity in human hepatocytes, but cannot predict efficacy maintenance in immunocompetent patients due to the immunodeficient background; complete non-human primate data (immunocompetent, HBV-infected, long-term follow-up) is currently absent, representing the weakest link in the evidence chain between preclinical and clinical settings. In contrast, the contemporaneously published *Pcsk9* programs (Cappelluti et al., Nature 2024; Tremblay et al., Nature Medicine 2025) provided durability data exceeding 12 months in immunocompetent models [9, 10]. This evidence gradient difference has direct guidance value for setting appropriate expectations when interpreting TUNE-401 Phase I results: the primary significance of Phase I is confirming safety and preliminary pharmacodynamics (cccDNA methylation density, HBsAg dynamics), not validating long-term efficacy.

##### Assessment of clinical design rationale

The Phase Ib trial conducted in New Zealand and Hong Kong employs an appropriate dose-escalation safety-first approach, with serum HBV DNA, HBsAg, and where feasible quantitative liver biopsy cccDNA as pharmacodynamic endpoints. Intravenous administration via Acuitas-formulated LNPs leverages their known hepatic tropism. The single-dose design aligns with the epigenetic ‘hit-and-run’ paradigm—however, the re-dosing strategy for patients with suboptimal initial response has not been clearly defined, representing a gap in the clinical protocol that needs to be addressed.

##### Key unanswered questions

First, what is the minimum methylation density on HBV cccDNA required for durable suppression, and can circulating cell-free DNA be quantified as a non-invasive biomarker? Second, how does integrated HBV DNA—whose copy number exceeds cccDNA by 10–1000-fold in many chronically infected livers—respond to the epigenetic editor, and does silencing of integrated HBV independently contribute to functional cure endpoints?

#### The PCSK9 case: dual preclinical validation of durability and reversibility

##### Strategic value as the core argument for reversibility

The *Pcsk9* preclinical data published in Nature Medicine in 2025 [9] merit analysis beyond their efficacy metrics, as they constitute the most rigorous available demonstration of two attributes of core significance to the therapeutic characteristics of epigenetic editing—annualized durability and in vivo reversibility—and have important mechanistic support implications for both Criterion 1 and Criterion 3.

##### Detailed assessment of durability evidence

Approximately 90% reduction in circulating *Pcsk9* protein in cynomolgus monkeys maintained for at least 3 months, combined with ≥ 12 months of durability in humanized *Pcsk9* transgenic mice, establishes a compelling efficacy case. Critically, persistence through liver regeneration induced by partial hepatectomy (involving 2–3 rounds of hepatocyte division completed within 5–7 days post-surgery) provides direct in vivo evidence that the KRAB-DNMT3A-DNMT3L tripartite effector can establish sufficient methylation density and H3K9me3 co-deposition for faithful replication-coupled DNMT1 maintenance. Quantitative methylation data (greater than 80% CpG methylation at the targeted *Pcsk9* promoter) exceeds the empirical stability threshold discussed in Section Molecular determinants of epigenetic memory stability: quantitative kinetics and tissue context, providing mechanistically consistent support for the durability observations.

##### Assessment and limitations of reversibility evidence

In mice silenced for more than 173 days, dCas9-TET1 activator administration restored approximately 90% of baseline *Pcsk9* expression [9], representing the first in vivo proof-of-concept of pharmacological reversal of therapeutically established epigenetic silencing. However, the translational gap between this mouse experiment and clinical reversibility remains substantial across three dimensions. First, the activator is delivered via the same LNP route as the silencing agent, presupposing that patients designated for reversal can receive a second LNP administration without immunological complications—an assumption not yet validated. Second, although *Pcsk9* recovery was assessed at multiple timepoints, with plasma levels maintaining near baseline throughout an 8-week observation window, the observation window remains limited: whether re-silencing occurs after 8 weeks has not been assessed, and the long-term in vivo stability of the reactivated state remains unknown. Third, scenarios actually triggering clinical reversal decisions—such as unexpected adverse effects from sustained LDL lowering (e.g., hemorrhagic stroke risk in specific patient subpopulations)—have not been modeled, and the infrastructure required for clinical implementation of reversibility, including validated biomarkers for monitoring silencing drift, defined clinical indications for activator dosing, and safety data for activating editors themselves, is currently entirely absent.

##### Competitive landscape and clinical value proposition

nChroma Bio’s PCSK9 program and Scribe Therapeutics’ STX-1150 face a competitive landscape where existing PCSK9 inhibitors (evolocumab, inclisiran) can already achieve durable LDL reduction with established safety profiles. Scribe Therapeutics reported STX-1150 data at the 2025 American Heart Association Scientific Sessions: durable ≥ 50% LDL-C reduction following single injection maintained in non-human primates for more than 515 days, with peak reduction reported as high as 67%. This competitive landscape reflects a pattern common in early platform validation: multiple companies converging on PCSK9 as a shared proof-of-concept target, with differentiation ultimately depending on safety profiles and delivery systems rather than target selection. The clinical value proposition of epigenetic editing in the PCSK9 indication critically hinges on demonstrating superior adherence outcomes from a single administration versus inclisiran’s twice-yearly dosing regimen, alongside an acceptable safety profile comparable to established PCSK9 inhibitors—requirements that will demand both sufficiently powered Phase III trials and robust real-world evidence to satisfy.

### Criterion 2 validation: tissue-selective delivery beyond the liver

#### EPI-321 and FSHD: first-in-human validation of skeletal muscle targeting

##### Technical basis and mechanistic compatibility

EPI-321 targets the D4Z4 repeat sequence on chromosome 4q35, suppressing de-repressed DUX4 transcription by restoring hypermethylation at this pathologically hypomethylated locus in FSHD type 1 patients [16, 124]. Use of the AAVrh74 capsid leverages muscle tropism demonstrated in Duchenne muscular dystrophy trials, enabling systemic delivery to skeletal muscle—a post-mitotic tissue context starkly different from the liver-focused programs described above, directly benchmarking against Criterion 2’s requirement of ‘target organ efficiency ≥ 50% (beyond the liver).’

FSHD represents an unusual epigenetic editing target: the pathogenic mechanism is itself epigenetic (D4Z4 hypomethylation leading to DUX4 de-repression) rather than sequence-based, making epigenetic repair conceptually more direct than genome editing. The post-mitotic nature of mature skeletal muscle fibers has dual significance: on one hand an advantage (established methylation without replication dilution); on the other a challenge—skeletal muscle contains a minority population of mitotically active satellite cells essential for muscle regeneration, and in myopathy contexts (where damage repair frequently occurs), silencing must be maintained through satellite cell division and differentiation to ensure durability (the tradeoff between CHARM and CRISPRoff discussed in Section Molecular determinants of epigenetic memory stability: quantitative kinetics and tissue context applies directly here).

##### Preclinical evidence and limitations

Preclinical studies demonstrate potent suppression of DUX4 expression and muscle tissue protection [124], but as of the time of writing this review, complete peer-reviewed NHP data with quantitative D4Z4 methylation endpoints had not been publicly published. The AAVrh74 vector used in EPI-321 comes from Sarepta Therapeutics’ SRP-9003 program (limb-girdle muscular dystrophy), providing cross-program safety information—albeit with different doses and different transgene payloads.

##### Key unanswered questions

First, can the GEMS (Genome Epigenetic Modification System) system achieve sufficient methylation density at D4Z4 to withstand satellite cell-mediated muscle regeneration in FSHD patients—whose satellite cell pool may already be depleted by chronic DUX4-mediated myotoxicity? Second, what is the minimum effective DUX4 suppression threshold required for clinical benefit (prevention of disease progression, functional improvement, or both)? Third, how will immune responses to the AAVrh74 capsid affect the feasibility of re-dosing in patients with insufficient initial response?

#### CHARM and prion disease: proof-of-concept for whole-brain delivery

Neumann et al.‘s CHARM system, published in Science in 2024 [14], represents a breakthrough in compact epigenetic editor design for neurological applications, providing the strongest preclinical evidence to date for both Criterion 1 (durability) and Criterion 2 (brain delivery competence). Systemic delivery via AAV-PHP.eB achieved more than 80% whole-brain PrP expression knockdown, far exceeding the minimum knockdown threshold needed to trigger therapeutic effects. The researchers reported that CHARM can function even when administered after symptom onset in prion-infected mice, suggesting potential efficacy in established disease [14].

The potential applicability of CHARM to broader neurodegenerative diseases (Huntington’s disease, Parkinson’s disease, ALS, Alzheimer’s disease) is attractive, but each indication requires specific validation targeting the expression characteristics, cell type distribution, and minimum effective silencing threshold of the specific target gene. It is particularly important to note that while whole-brain delivery efficiency of AAV-PHP.eB is well validated in mice, its efficiency is known to be significantly lower in non-human primates—a species difference with direct implications for planning CHARM’s clinical translation pathway [104, 105].

### Criterion 3 validation: OTX-2002 and the first human evidence for epigenetic target engagement

Omega Therapeutics’ OTX-2002 represents the first epigenomic controller to complete Phase I dose escalation, with its clinical trajectory providing the first signals from humans regarding oncological applications of epigenetic editing—strategic significance exceeding the numbers themselves. The MYCHELANGELO™ I trial completed six dose cohorts (0.02 to 0.3 mg/kg) in 24 patients, with principal findings including: highly specific target engagement, robust dose-dependent increases in MYC DNA methylation (detectable in cell-free DNA), durable MYC expression downregulation, and an acceptable safety profile with infusion-related reactions as the most common adverse event [22]. Disease control rate was 50% in HCC patients with evaluable efficacy.

Several key observations merit deep analysis, particularly in the context of the company’s subsequent suspension of clinical development. From an evidence quality perspective, the primary purpose of Phase I dose escalation design is safety assessment rather than efficacy validation; a 50% disease control rate in HCC—compared with sorafenib (first-line objective response rate approximately 2% by RECIST v1.1, SHARP trial) [125] and atezolizumab plus bevacizumab (objective response rate approximately 27% by RECIST v1.1 and approximately 33% by mRECIST, IMbrave150 trial primary analysis) [126]—is neither clearly superior nor clearly ineffective, falling in a gray zone requiring Phase II randomized data to interpret. Omega’s commercial decision—to suspend development and seek partners—likely reflects capital allocation and development prioritization considerations more than a negation of epigenetic editing mechanism validity—this distinction is crucial for avoiding misinterpretation of commercial decisions as scientific negations.

A more deeply critical analysis from the disease selection perspective: MYC is upregulated in more than 50% of human cancers but is also a core regulator of normal cell proliferation. The therapeutic window of epigenetic silencing rather than complete elimination—wherein a certain proportional reduction in MYC expression selectively affects tumors rather than normal tissue—has not been quantitatively defined, representing an inherent biological challenge with this target, independent of the delivery platform. From a competitive landscape perspective, the HCC field in 2024 already had the atezolizumab+bevacizumab combination regimen as first-line standard (ORR approximately 27% by RECIST v1.1; approximately 33% by mRECIST in the IMbrave150 trial), significantly superior to sorafenib (ORR approximately 2% by RECIST v1.1 in the SHARP trial), setting a higher bar for differentiation of new therapies. A 50% disease control rate needs to be interpreted in this context rather than merely compared with historical controls. Additionally, median follow-up time and cohort size in Phase I data are key contextual factors for interpreting ‘durability’ signals; currently available public information is insufficient to assess this dimension, and this information gap itself constitutes an important component of the analysis.

Overall, OTX-2002 provides human proof-of-concept that ‘epigenetic editors can reach tumor targets and produce detectable methylation changes’—of significant importance for Criterion 3 fulfillment—but whether this can translate into clinical benefit requires larger, controlled studies to determine.

It is noteworthy that another recently registered clinical trial, CRMA-1001 (NCT07200193, currently recruiting), further expands the clinical landscape of epigenetic editing. This trial represents another human validation project with preset pharmacodynamic endpoints following OTX-2002, TUNE-401, and EPI-321; its specific targets and delivery strategies await publication of peer-reviewed data; within the three maturity criteria framework, its Criterion 3 (initiation of human trials) has been satisfied, while the status of Criterion 1 and Criterion 2 preclinical accumulation awaits subsequent data support.

### Critical comparative assessment of the emerging clinical landscape

#### China’s epigenetic editing landscape: discussion basis and tiered assessment

China merits separate discussion for two objective reasons: first, China has become the world’s second-largest gene therapy clinical trial market; second, China has an independent regulatory framework from the FDA and EMA—the National Medical Products Administration (NMPA)—whose approval standards and data requirements differ substantially from Western systems, directly affecting how international readers interpret Chinese clinical data. Within this landscape, the most directly relevant advance to epigenetic editing comes from Shanghai Epigenic Therapeutics, whose proprietary EPIREG^®^ platform—a dCas9-based epigenetic editing system that achieves targeted gene silencing through synergistic DNA methylation and histone modification without relying on DNA cleavage—represents China’s most mature true epigenetic editing program to date. EPI-003 delivers mRNA encoding epigenetic regulatory proteins and guide RNAs targeting both HBV cccDNA and integrated HBV DNA via LNPs, received CTA approval from New Zealand’s Medsafe in November 2024, followed by IND filing clearance from NMPA, and is currently conducting multicenter Phase I trials in New Zealand, Australia, and Hong Kong [127]. In September 2025, Epigenic completed a $60 million Series B financing to accelerate both programs. By the three criteria assessment, EPI-003 has met Criterion 3 (human trials initiated) and has good Criterion 1 preclinical evidence, but quantitative methylation durability data in immunocompetent models—a critical evidence gap similar to that facing TUNE-401—remains to be generated.

At the regulatory ecosystem level, NMPA registered 115 cell and gene therapy clinical trials in 2024—a 42% increase from 2023. This quantitative growth reflects accelerated approval speed, but quantity does not equate to quality improvement: some early trials in the historical record of Chinese gene therapy clinical trials had regulatory data requirements below FDA/EMA standards, and researchers should note this context when citing Chinese clinical data to assess its international transferability. At the global collaboration level, policy changes in September 2024 allowing foreign enterprises to participate in human stem cell and gene diagnosis and treatment technology development and application in four free trade zones, and the Shanghai Gene Therapy Action Plan (2023–2025) providing up to RMB 30 million in financial support, have created new opportunities for both domestic and international participants. However, intellectual property protection and data sharing mechanisms remain substantive barriers to cross-border collaboration.

### Unresolved clinical translation bottlenecks

Despite the encouraging advances described above, several fundamental challenges continue to impede broader application of epigenetic editing technology: the validation gap for long-term stability in post-mitotic cells (cardiomyocytes, skeletal muscle fibers, mature neurons) (detailed in Section The non-dividing cell problem: a critical translational gap); multi-dimensional immunogenicity challenges including pre-existing anti-Cas9 immunity and high-dose AAV-associated toxicity; manufacturing cost and pricing creating access barriers (detailed in Section Technology integration trends and key challenges); and the lack of monitoring and intervention infrastructure required to make reversibility clinically actionable (detailed in Section Multi-layered safety mechanisms of epigenetic editing).

## Perspectives and outlook

The maturation of epigenetic editing as a clinical therapeutic modality requires critical evaluation of its broader implications, emerging applications, and remaining challenges. This section synthesizes the preceding advances into a coherent framework, objectively assesses the multi-layered safety mechanisms that distinguish epigenetic editing from permanent genome editing, examines broader applications from programmable cell memory to aging intervention, and candidly acknowledges current limitations.

### Multi-layered safety mechanisms of epigenetic editing

The safety assurance of epigenetic editing can be understood through five interrelated tiers, with the following supplementation of quantitative data to enhance accurate understanding of the actual effectiveness of mechanisms at each tier.

The first tier is entry safety, achieved through AI-optimized high-specificity DNA-binding domains and sgRNA design for precise targeting. Nuñez et al.‘s study showed that CRISPRoff induced less than 2% genome-wide increase in DNA methylation, with only 1–3 off-target transcripts altered per experiment [11]. It is noteworthy that while this improvement is substantial compared with traditional Cas9 nucleases (which can produce dozens to hundreds of off-target sites) [128], KRAB effector domain-mediated transcriptional repression may also cause indirect expression changes in off-target genes—a potential indirect effect requiring assessment through whole-transcriptome monitoring.

The second tier is execution safety, where epigenetic editing circumvents the genotoxicity risks of traditional genome editing at the DNA sequence integrity level. Kosicki et al. warned that Cas9 nuclease-induced double-strand breaks can lead to large deletions extending thousands of bases and complex genomic rearrangements [2], risks completely avoided by epigenetic editing; chromothripsis occurring at approximately 1–2% frequency [3] is likewise circumvented by epigenetic editing. However, a critical distinction must be made: epigenetic editing can still cause indirect off-target expression changes in non-targeted genes through effector domain-mediated transcriptional regulation (as noted in the first tier)—these are regulatory-level off-target effects rather than DNA sequence damage; the two types of effects have fundamentally different safety implications and reversibility.

The third tier is targeting safety, where tissue-selective delivery platforms confine editor activity to the intended target organ, reducing systemic off-target exposure. SORT technology exemplifies this principle by redirecting delivery from liver to lung (reducing liver uptake approximately 100-fold) or spleen through addition of a fifth lipid component [93], thereby limiting editor expression to the desired tissue compartment; however, organ immune activation data for non-hepatic targeting LNPs remains insufficient, and this safety feature requires data accumulation across more indications.

The fourth tier is the reversibility mechanism, where the CRISPRoff/CRISPRon paired system makes correction of adverse outcomes possible. nChroma Bio’s *Pcsk9* study confirmed that after more than 173 days of silencing, targeted epigenetic activator treatment restored mouse *Pcsk9* expression levels to near baseline within 8 weeks [9]. However, the time course of complete recovery, whether re-silencing occurs, and the absence of corresponding data in non-human primates all create substantial uncertainties regarding clinical reversibility implementation (detailed assessment in Section The PCSK9 case: dual preclinical validation of durability and Reversibility). The validated biomarkers and activator dosing protocols required for reversibility implementation should be developed in parallel with efficacy programs rather than initiated after adverse events occur.

**Fig. 5:**
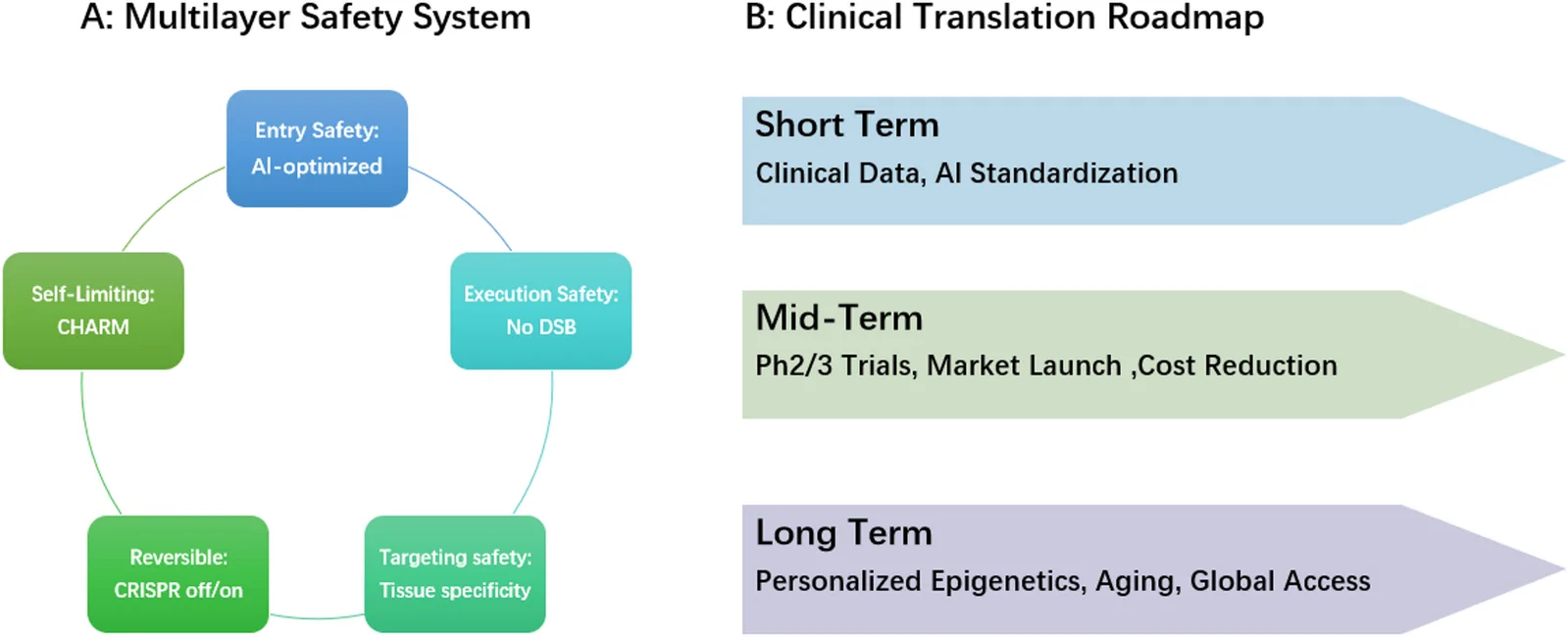
Multilayer safety architecture and clinical translation roadmap for epigenetic editing. **A** Five-layer safety system integrating AI-optimized entry, DSB-free execution, organ-selective targeting, CRISPRoff/on reversibility, and CHARM self-limiting mechanisms. **B** Clinical translation phases from short-term data generation through mid-term market launch to long-term personalized applications. DSB: double-strand break; CHARM: compact histone-associated repressor of methylation

The fifth tier is self-limitation, where CHARM’s kinetically tuned design enables the editor to automatically silence its own expression after completing target gene silencing, circumventing chronic expression risks [14]; transgene expression in mouse brain automatically decreased to background levels within 4–8 weeks, but whether this mechanism is equally effective in high-turnover cells has not been tested. The multilayer safety system and clinical translation roadmap for epigenetic editing are illustrated in Fig. 5.

### Broader implications and applications

Epigenetic editing transcends the paradigm of single-gene therapy, offering unique capabilities across multiple domains. As a programmable cell memory tool, epigenetic editors can write durable, heritable memory states that persist after editor degradation—providing significant advantages over traditional synthetic biology circuits requiring continuous expression [129, 130]. This characteristic has broad application potential in cell therapy safety switches and stable synthetic gene circuits. Tune Therapeutics has demonstrated that CAR T cells can be enhanced in proliferation, persistence, and anti-tumor activity through in vitro epigenetic modification; nChroma Bio (formerly Chroma Medicine) has demonstrated data showing multiplex epigenetic editing silences TCR and MHC genes to enhance allogeneic CAR T cell therapy [131].

For complex diseases involving network dysregulation, multiplex epigenetic editing enables synergistic multi-target intervention: recent studies demonstrated that multiplex CRISPRoff sensitizes glioblastoma to chemotherapy through stable MGMT suppression maintained for more than 8 months [132], demonstrating the translational value of epigenetic memory in oncology. In the aging intervention field, related epigenetic reprogramming approaches (mechanistically related to targeted epigenetic editing) have shown promise: in progeria mouse models, periodic OSKM (*Oct4*, *Sox2*, *Klf4*, and *c-Myc*—the Yamanaka reprogramming factors) expression extended median lifespan by 33% in progeria mouse models [133], with epigenetic clock analyses confirming concurrent reversal of epigenomic age [134].

At the personalized precision medicine level, chromatin accessibility significantly affects editing efficiency, with nucleosome occupancy strongly negatively correlated with CRISPR activity [135], emphasizing the importance of individualized approaches. Deep learning models such as eDICE can predict individual-specific epigenetic variation for epigenomic imputation [136]. Pharmacodynamic monitoring of therapeutic epigenetic editing requires a tiered, indication-specific strategy rather than a single universal approach. For hepatic targets, circulating protein biomarkers—such as serum PCSK9 levels and HBsAg titers—provide clinically established, quantitative surrogates for transcriptional silencing that are already incorporated as primary pharmacodynamic endpoints in current Phase I trials [15, 22]. For tissues accessible to biopsy, targeted bisulfite sequencing or pyrosequencing of locus-specific CpG methylation density at the edited genomic region serves as the most direct quantitative readout of editing efficacy, and has been validated as the primary pharmacodynamic endpoint in leading preclinical programs [9, 10]. For neurological applications where repeated tissue sampling is impractical, cerebrospinal fluid biomarkers or imaging-based functional surrogates may be required, though none have yet been validated specifically for epigenetic editing contexts. While methylation-based liquid biopsy technologies have shown promise for cancer detection, their performance is highly dependent on cancer type and the extent of tumor DNA shedding into the bloodstream; extension of this approach to monitor therapeutically established epigenetic edits introduces additional uncertainties, as edited methylation signatures would need to be present at sufficient quantity, sufficiently stable in circulation, and distinguishable from background methylation variation to be clinically actionable. The development of minimally invasive monitoring tools validated specifically for edited methylation signatures—rather than repurposing cancer detection technology—represents an important unmet need for the field.

### Technology integration trends and key challenges

The clinical translation timeline can be predicted in three phases. Short-term (2025–2027), first clinical trials such as TUNE-401 will provide critical data, AI design tools will become further standardized, and the first in vivo data on human epigenetic silencing durability will validate or challenge existing mechanistic models. Medium-term (2027–2030), multiple indications will enter clinical trials, individualized epigenetic therapy platforms are expected to be established, and delivery platform optimization based on head-to-head comparisons will establish optimal strategies for each indication. Long-term (beyond 2030), epigenetic editing is expected to become one of the mainstream gene therapy modalities, deeply integrated with regenerative medicine and aging intervention. A dimension that merits explicit acknowledgment in this long-term outlook is the potential expansion from single-locus editing—which constitutes the exclusive focus of all current clinical programs—toward multi-locus or broader regional epigenome editing. Current therapeutic designs target one or a small number of discrete genomic loci, reflecting both the precision requirements of early clinical development and the technical constraints of current targeting modules. However, many disease-relevant epigenetic alterations are not confined to single loci: cancer epigenomes exhibit broad regional hypomethylation and focal hypermethylation across hundreds of CpG islands; neurodegenerative diseases involve progressive, genome-wide epigenetic drift; and complex polygenic disorders may require coordinated silencing or activation across multiple loci simultaneously. As AI-designed targeting modules become smaller and more programmable, and as multiplexed delivery systems mature, multi-locus epigenome editing may become technically feasible within this decade. However, two prerequisites must be met before broader regional editing can be responsibly translated: first, targeting specificity must be rigorously validated at genome-wide resolution to ensure that expanded editing footprints do not perturb non-target loci; second, safety monitoring frameworks capable of detecting regional epigenetic dysregulation—potentially including unintended silencing of tumor suppressor genes or activation of oncogenes—must be established prospectively. AI-driven approaches to predict off-target regional effects and design spatially constrained editing systems may offer important strategies toward this goal, but represent a frontier that current experimental and computational tools are only beginning to address.

However, several key challenges remain to be overcome. At the scientific level, epigenetic stability in post-mitotic cells across therapeutic time scales is the core bottleneck constraining broader applications, discussed specifically in Section The non-dividing cell problem: a critical translational gap. An asymmetry not covered in Section The non-dividing cell problem: a critical translational gap merits supplementation here: activation-type applications face greater durability challenges than silencing-type applications—DNA methylation-mediated silencing can be faithfully maintained through DNMT1 during cell division, whereas activation-associated histone marks (H3K4me3, H3K27ac) lack equivalent self-reinforcing mechanisms and are subject to rapid turnover by demethylases and deacetylases [10, 137]. This means the three maturity criteria framework is currently validated primarily in silencing-type programs and cannot be directly extrapolated to activation-type programs; developing effector domain combinations capable of achieving durable activation is an important direction for next-generation editors. Beyond the silencing-activation asymmetry described above, efficacy differences across cell types and target genes represent an equally important but underappreciated limitation for therapeutic target selection. While CRISPRoff demonstrates broad efficacy across diverse genomic targets in dividing cells, certain targets remain recalcitrant to epigenetic repression, likely attributable to factors including CpG density, genomic context, and chromatin organization at the target locus. The challenge is particularly pronounced for activation-type epigenetic editing. A recent study employing two complementary approaches to induce expression of “silent” Mendelian genes (SMGs) in human dermal fibroblasts (HDFs) yielded instructive contrasting results: highly multiplexed CRISPRa transactivation achieved induction of 20/20 (100%) tested SMGs in HDFs, whereas transdifferentiation of HDFs directly to neurons yielded expression of only 193/516 (37.4%) of SMGs implicated in neurological disease [138]. These findings demonstrate that the choice of activation strategy and cell type context critically determines feasibility—CRISPRa transactivation outperformed transdifferentiation for neurological targets, but neither approach guarantees universal applicability across all target genes and cell types. These observations carry direct implications for the selection and feasibility of in vivo therapeutic targets in epigenetic editing: the assumption that any genomic locus is equally amenable to epigenetic activation regardless of cell type cannot be sustained by current evidence. For programs aiming to restore silenced gene expression in post-mitotic tissues—such as neurons or skeletal muscle fibers—preclinical target validation must include cell type-specific accessibility assessments, and AI-driven design approaches capable of predicting chromatin context-dependent activation efficiency may offer important strategies to guide target selection and improve the likelihood of clinical success. At the technical level, long-term stability of epigenetic marks in non-dividing cells remains a bottleneck (detailed in Section The non-dividing cell problem: a critical translational gap), and viral vector production costs of $3–7 million per batch constrain commercialization progress. At the translational level, regulatory differences between FDA and EMA—including different approval timelines, efficacy standards, and post-marketing requirements—add complexity to global development. At the business model level, gene therapy pricing of $2–4 million per patient creates severe access and equity challenges, with innovative outcome-based payment models being actively explored [139].

### The non-dividing cell problem: a critical translational gap

For broader application of epigenetic editing, the most important unresolved challenge is the behavior of epigenetic marks in permanently post-mitotic cells—cardiomyocytes, skeletal muscle fibers, and mature neurons—across the decades-long therapeutic time scales. Current durability evidence derives primarily from two complementary contexts: continuously dividing in vitro human cell lines (CRISPRoff: more than 450 divisions [11]) and in vivo liver (undergoing regeneration under experimental conditions [9, 10]). Both contexts involve cell division, making DNMT1-mediated maintenance methylation the primary stability mechanism. As systematically articulated in Section Molecular determinants of epigenetic memory stability: quantitative kinetics and tissue context, in post-mitotic cells, TET-mediated active demethylation is the primary threat to long-term stability, and resistance to it is highly dependent on cell type-specific TET activity levels.

For cardiac applications (potentially encompassing hypertrophic cardiomyopathy, arrhythmias, and heart failure), adult cardiomyocytes are permanently post-mitotic. Silencing pathogenic genes in cardiomyocytes (such as SCN5A gain-of-function mutations causing long QT syndrome) requires resisting active TET-mediated demethylation without any replication-dependent maintenance mechanism assistance. To date, no published study has demonstrated that epigenetic marks deposited by exogenous editors in adult cardiomyocytes can remain stable for years. The high metabolic activity and oxidative stress environment of cardiomyocytes may accelerate TET-mediated demethylation compared with neurons.

For skeletal muscle, the situation is heterogeneous: mature multinucleated muscle fibers are post-mitotic, but satellite cells (muscle stem cells) are mitotically active and essential for muscle repair in myopathy contexts where damage repair frequently occurs. Whether methylation marks deposited by CRISPRoff or CHARM at disease-associated loci can be faithfully maintained through satellite cell activation, proliferation, and differentiation into new muscle fibers is a key question for any muscular dystrophy application, directly affecting the credibility of EPI-321 efficacy predictions.

Addressing the non-dividing cell problem may require one of three approaches: first, developing epigenetic editors with enhanced H3K9me3/DNA methylation co-deposition capacity capable of exceeding the TET-mediated elimination rate in specific cell types—a challenge equally applicable to activation-type programs, where achieving durable activation is more difficult than achieving durable silencing due to the lack of self-reinforcing maintenance mechanisms for activating histone marks; second, integrating self-reinforcing elements to actively maintain epigenetic marks without dependence on DNA replication (analogous to PRC2 maintaining H3K27me3 through allosteric activation) [140]; third, accepting that periodic re-dosing may be required in post-mitotic contexts, fundamentally adjusting the ‘one-time treatment’ value proposition. This challenge deserves explicit acknowledgment and discussion in the design criteria for next-generation epigenetic editors targeting cardiac, muscle, and neurological applications.

## Conclusion

That epigenetic editing has evolved from a laboratory tool to a clinically validated therapeutic modality in just a few years is itself sufficiently remarkable. By leveraging the self-reinforcing interactions between H3K9me3 and DNA methylation, contemporary systems have established durable silencing that persists across hundreds of cell divisions and have completed proof-of-concept pharmacological reversal in animal models—this technical combination is the fundamental hallmark of the field’s maturation. The elegant solution of the CHARM system—hijacking endogenous methylation machinery through a tripartite fusion of zinc finger DNA-binding domain, DNMT3L C-terminal domain, and unmethylated histone H3 N-terminal tail decoy—demonstrates that deep mechanistic understanding can generate compact, clinically feasible architectures compatible with single-AAV delivery.

Artificial intelligence has accelerated this field across three dimensions, but its contributions must be accurately understood by tier. At the level of directly advancing clinical progress, deep learning platforms such as AGILE and COMET have transformed LNP optimization from empirical screening to systematic engineering, and machine learning-guided AAV capsid directed evolution has achieved tissue selectivity previously unattainable; at the level of optimized existing tools not yet entering clinical programs, structure prediction tools represented by AlphaFold3 have enabled rational design of protein–DNA interactions with unprecedented precision, generative models including RFdiffusion, and the 2025 breakthrough in programmable DNA-binding protein design have opened pathways to targeting modules smaller than 65 amino acids—bypassing the size, PAM requirements, and immunogenicity issues inherent to CRISPR-Cas systems; at the level of breakthrough potential with zero clinical precedent, de novo designed DNA-binding proteins represent a proof-of-concept milestone, but the journey from this scientific achievement to clinical application still spans years of development.

Assessed by the three quantifiable maturity criteria, the current evidence landscape presents a clear hierarchical structure: preclinical support for mechanistic durability is relatively robust—maintained for more than 3 months in cynomolgus monkeys, more than 12 months in humanized mice, combined with TUNE-401’s more than 550 days in human cell lines, and mechanistically validated through liver regeneration experiments; delivery competence has matured for liver, is in early clinical validation stages for skeletal muscle and whole brain, and human biopsy methylation data for non-hepatic organs remains absent; clinical validation is preliminarily satisfied with TUNE-401 and EPI-321 Phase I initiations, but human in vivo durability and pharmacodynamic endpoint data have not yet matured. Simultaneous satisfaction of all three criteria in humans remains the outstanding core task of this field. Clinical implementation of reversibility depends on monitoring and intervention infrastructure established in parallel with efficacy programs (detailed in Section Multi-layered safety mechanisms of epigenetic editing), which is the prerequisite for transforming it from an animal experimental concept to clinical practice assurance.

However, while candidly acknowledging these advances, several key uncertainties must be explicitly stated. The fundamental question of long-term epigenetic stability in humans—across decades rather than the months of preclinical observation—cannot be answered by existing data. The assumption that H3K9me3/DNA methylation co-deposition will remain indefinitely stable in human somatic cells relies on mechanistic reasoning and medium-term animal data; clinical programs themselves will provide definitive evidence, making patient monitoring through methylation liquid biopsies not only a requirement of clinical prudence but a scientific necessity. The validation gap for long-term epigenetic stability in non-dividing cells (detailed in Section The non-dividing cell problem: a critical translational gap) remains the core unresolved issue constraining realization of this therapy’s complete potential.

From a therapeutic access perspective, equity challenges created by manufacturing costs and pricing cannot be solved by scientific breakthroughs alone; outcome-based payment models and international regulatory coordination are necessary prerequisites for ensuring this emerging therapy truly benefits patients.

With the above caveats clearly stated, the trajectory of epigenetic editing toward clinical impact is compelling. The convergence of mechanistic understanding, computational design, and delivery engineering has transformed this field from laboratory concept to clinical reality within a decade. What the coming years will determine is not whether epigenetic editing can work, but at what breadth of application, what intensity of durability, and what degree of equity this will be realized.

## Data Availability

No datasets were generated or analysed during the current study.

## Abbreviations

AAV: Adeno-associated virus
ALS: Amyotrophic lateral sclerosis
ALT: Alanine aminotransferase
AST: Aspartate aminotransferase
AUC: Area under the curve
CAR T: Chimeric antigen receptor T cell
cccDNA: covalently closed circular DNA
CNS: Central nervous system
CRISPR: Clustered regularly interspaced short palindromic repeats
CRISPRa: CRISPR activation
CRISPRi: CRISPR interference
Cas9: CRISPR-associated protein 9
dCas9: catalytically inactive Cas9
DBP: DNA-binding protein
DNMT: DNA methyltransferase
DNMT1: DNA methyltransferase 1
DNMT3A: DNA methyltransferase 3 alpha
DNMT3L: DNA methyltransferase 3-like
EMA: European Medicines Agency
EPIREG: Epigenetic regulation platform (proprietary platform of Epigenic Therapeutics)
FDA: U.S. Food and Drug Administration
FSHD: Facioscapulohumeral muscular dystrophy
GDT: Global distance test
GEMS: Genome Epigenetic Modification System
HBV: Hepatitis B virus
HBsAg: Hepatitis B surface antigen
HBcAg: Hepatitis B core antigen
HCC: Hepatocellular carcinoma
HDF: Human dermal fibroblast
H3K9me3: Histone H3 lysine 9 trimethylation
H3K4me1/me2: Histone H3 lysine 4 mono/demethylation
H3K27me3: Histone H3 lysine 27 trimethylation
HP1: Heterochromatin protein 1
iPSC: Induced pluripotent stem cell
KRAB: Krüppel-associated box
LDL: Low-density lipoprotein
LDL-C: Low-density lipoprotein cholesterol
LNP: Lipid nanoparticle
MHC: Major histocompatibility complex
mRNA: messenger RNA
NHP: Non-human primate
NK: Natural killer
NMPA: National Medical Products Administration
PAM: Protospacer adjacent motif
PCSK9: Proprotein convertase subtilisin/kexin type 9
PDB: Protein Data Bank
pLDDT: predicted local distance difference test
PRC2: Polycomb repressive complex
2RMSD: root mean square deviation
RNP: Ribonucleoprotein
RVD: Repeat variable di-residue
sgRNA: single guide RNA
siRNA: small interfering RNA
SMG: Silent Mendelian gene
SORT: Selective organ targeting
TCR: T cell receptor
TALE: Transcription activator-like effector
TDG: Thymine-DNA glycosylase
TET: Ten-eleven translocation
TEMPO: Tune Therapeutics Epigenetic Modulation Platform
UTR: Untranslated region
ZFP: Zinc finger protein
5caC: 5-carboxylcytosine
5fC: 5-formylcytosine
5hmC: 5-hydroxymethylcytosine
5mC: 5-methylcytosine
